# Identification and evaluation of bioactive compounds from *Azadirachta indica* as potential inhibitors of DENV-2 capsid protein: An integrative study utilizing network pharmacology, molecular docking, molecular dynamics simulations, and machine learning techniques

**DOI:** 10.1016/j.heliyon.2025.e42594

**Published:** 2025-02-12

**Authors:** Md. Ahad Ali Khan, Md. Nazmul Hasan Zilani, Mahedi Hasan, Nahid Hasan

**Affiliations:** a Department of Pharmacy, Manarat International University, Dhaka, Bangladesh; b Department of Pharmacy, Jashore University of Science and Technology, Jashore, Bangladesh

**Keywords:** Anti-dengue, *Azadirachta indica*, Capsid protein, DENV-2, Molecular docking, Molecular dynamics simulation, Network pharmacology

## Abstract

**Background:**

Dengue fever is a viral disease caused by the dengue flavivirus and transmitted through mosquito bites in humans. According to the World Health Organization, severe dengue causes approximately 40,000 deaths annually, and nearly 4 billion people are at risk of dengue infection. The urgent need for effective treatments against the dengue virus has led to extensive research on potential bioactive compounds.

**Objective:**

In this study, we utilized a network pharmacology approach to identify the DENV-2 capsid protein as an appropriate target for intervention. Subsequently, we selected a library of 537 phytochemicals derived from *Azadirachta indica* (Family: Meliaceae), known for their anti-dengue properties, to explore potential inhibitors of this protein.

**Methods:**

The compound library was subjected to molecular docking to the capsid protein to identify potent inhibitors with high binding affinity. We selected 81 hits based on a thorough analysis of their binding affinities, particularly those exhibiting higher binding energy than the established inhibitor ST-148. After evaluating their binding characteristics, we identified two top-scored compounds and subjected them to molecular dynamics simulations to assess their stability and binding properties. Additionally, we predicted ADMET properties using *in silico* methods.

**Results:**

One of the inhibitors, [(5S,7R,8R,9R,10R,13R,17R)-17-[(2R)-2-hydroxy-5-oxo-2H-furan-4-yl]-4,4,8,10,13-pentamethyl-3-oxo-5,6,7,9,11,12,16,17-octahydrocyclopenta[a]phenanthren-7-yl] acetate (AI-59), showed the highest binding affinity at −10.4 kcal/mol. Another compound, epoxy-nimonol (AI-181), demonstrated the highest number of H-bonds with a binding affinity score of −9.5 kcal/mol. During molecular dynamics simulation studies, both compounds have exhibited noteworthy outcomes. Through molecular mechanics employing Generalized Born surface area (MM/GBSA) calculations, AI-59 and AI-181 displayed negative ΔG_bind scores of −74.99 and −83.91 kcal/mol, respectively.

**Conclusion:**

The hit compounds identified in the present investigation hold the potential for developing drugs targeting dengue virus infections. Furthermore, the knowledge gathered from this study serves as a foundation for the structure- or ligand-based exploration of anti-dengue compounds.

## Highlights

• The study employs an innovative network pharmacology approach to drug discovery.
• *In silico* study validated the anti-dengue potential of *Azadirachta indica* compounds.
• Natural products showed superior molecular recognition capabilities.
• Identified inhibitors showed strong binding affinities with the DENV-2 capsid protein.
• AI-59 and AI-181 are promising lead compounds for anti-dengue drug development.

## Graphical abstract

**Figure:**
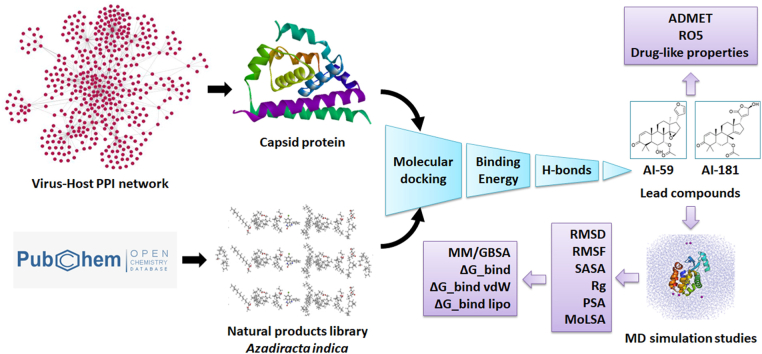

## 1 Introduction

Dengue fever is a viral illness caused by the dengue virus, which belongs to the flavivirus family [1,2]. The virus is transmitted through the bite of infected *Aedes* mosquitoes and is commonly found in tropical and subtropical regions [3,4]. The dengue virus has four different serotypes: DENV-1, DENV-2, DENV-3, and DENV-4, and it can infect people in any of these four forms. The most common symptoms of dengue fever include high fever, headache, and muscle pain. However, in many cases, the disease may be asymptomatic or cause only mild fever, which can be indistinguishable from other viral infections. In severe cases, the infection can progress to dengue hemorrhagic fever (DHF) or dengue shock syndrome [5,6].

In recent decades, global incidence rates of dengue fever have significantly escalated, affecting approximately 90 countries across various regions, including Africa, the Americas, Eastern Mediterranean, Southeast Asia, and Western Pacific. Current estimates suggest that about fifty percent of the global population remains susceptible to dengue infections [7]. According to the World Health Organization (WHO), between January and October 2024, there were 13,310,978 reported dengue cases worldwide, comprised of 7,052,484 confirmed cases, 46,339 severe cases, and 9,683 fatalities. The WHO Region of the Americas reported a staggering 12,457,099 confirmed cases, with 7,551 deaths. In contrast, Southeast Asian countries documented 435,525 cases with 1,638 deaths. Notably, Indonesia reported the highest incidence in 2024, with 210,644 cases, followed by Thailand and Bangladesh [8].

Bangladesh is highly susceptible to severe dengue fever outbreaks due to its climate and urbanization, which promote the spread of *Aedes* mosquitoes, the primary virus vectors [9]. As of December 25, 2024, Bangladesh's Directorate General of Health Services (DGHS) confirmed 100,558 laboratory-confirmed dengue cases and 565 deaths [10], illustrating a threefold increase compared to the same timeframe in the previous year. This trend underscores the escalating public health crisis associated with dengue transmission. Despite the increasing number and severity of dengue infections each year, there is no specific treatment available, and vector control, the primary strategy to combat the spread of dengue infections [6].

Research efforts are focused on discovering effective antiviral therapeutics to alleviate dengue symptoms. While certain compounds have shown promise in inhibiting dengue virus replication, none have demonstrated clinical efficacy against the viral infections [[11], [12], [13], [14], [15], [16], [17], [18], [19], [20]]. Consequently, the discovery of novel lead compounds for anti-dengue drugs is essential. The current lead discovery process relies heavily on high-throughput screening (HTS) methods, necessitating comprehensive compound libraries for evaluation. For instance, the identification of promising antiviral candidates, such as ST-148, required screening nearly 200,000 compounds [21], while VGTI-A3 was derived from screening 5,600 compounds. Despite high antiviral activity, VGTI-A3 required the synthesis of various analogs to enhance solubility, leading to the isolation of the new lead VGTI-A3-03 [22].

Traditional bioassay methods for detecting potential inhibitors are often resource-intensive and time-consuming [23], prompting the integration of artificial intelligence (AI) into drug discovery processes. AI facilitates rapid identification of viable therapeutic agents, leveraging extensive datasets to enhance predictive accuracy [24]. For drug development, AI-generated molecules have demonstrated an 80–90 % success rate in Phase I trials, with a subsequent 40 % success rate in Phase II based on limited datasets [25]. AI-driven computational simulation techniques optimize drug candidate selection, thereby reducing both research costs and experimental efforts [26]. Machine learning approaches, including molecular docking and ADMET predictions, are increasingly applied across various stages of drug development, with recent studies emphasizing potential inhibitors targeting dengue virus proteins [[27], [28], [29]].

The present study was carried out with the following objectives: (i) to investigate dengue virus proteins using a network pharmacology approach to identify a suitable receptor; (ii) to compile a library of bioactive compounds based on previous research demonstrating their efficacy against the dengue virus; (iii) to identify promising leads for anti-dengue drug development through molecular docking and molecular dynamics simulation studies; and (iv) to evaluate the drug-likeness, pharmacokinetics, and toxicological profiles of the lead compounds.

Receptor target identification is a crucial phase in the drug discovery pipeline, requiring a thorough compilation of biomedical research data that clarifies the roles of receptor targets in infectious processes and their potential for therapeutic intervention [30]. This study utilizes a network pharmacology framework to integrate protein-protein interaction data between the dengue virus and the human host, thereby shedding light on their dynamic relationship [31]. We have identified the capsid protein as a primary target for further investigation. This structural protein plays a vital role in the viral life cycle by encapsulating viral RNA within a nucleocapsid and facilitating the assembly of the mature virus particle. The precise encapsidation of the RNA genome by the dengue virus capsid protein leads to the formation of a circular nucleocapsid containing a single strand of RNA [[32], [33], [34], [35]].

For virtual screening, we developed a compound library inspired by the previous research of Parida, Upadhyay, and Jana, which demonstrated that an aqueous extract from the leaves of *Azadirachta indica* (AI) effectively inhibits DENV-2 replication in both *in vitro* and *in vivo* models [36]. Commonly known as the ‘neem' tree, *A. indica* is a well-known medicinal plant in South Asia and Africa, recognized for its ethnomedicinal uses in treating various health conditions [37]. In constructing this library, we have opted for plant secondary metabolites due to their remarkable macromolecular recognition capabilities, offering significant potential for targeted screening against specific protein receptors [38].

The study utilized an X-ray crystal structure of the DENV-2 capsid protein complexed with the inhibitor ST-148 (PDB ID: 6vg5) for molecular docking of *A. indica* compounds into the binding pocket of ST-148 [21]. Binding affinity and modes of interactions were compared between the phytochemicals and ST-148, while molecular dynamics simulations assessed the binding stability of the compounds. We leveraged machine learning tools to analyze the physicochemical properties of the compounds, their drug-likeliness, and ADMET profiles.

## 2 Materials and methods

### 2.1 Virus-host protein-protein interaction network

Six advanced bioinformatics tools Virusmentha [39], VirHostNet3.0 [40], Viruses. STRING [41], HPIDB3.0 [42], DenvInt [43], and DenHunt [44] were explored to obtain protein-protein interaction (PPI) networks for dengue virus. From these networks only PPI including dengue virus and human proteins were combined to build a network of Dengue-Human protein-protein interactions using Cytoscape 3.9.1 software [45]. The construction of the PPI network was tailored carefully to the ‘*Homo sapiens*' context, and criteria for evaluating the confidence level in interactions among target proteins were precisely adjusted to ensure high reliability. In this detailed network visualization, individual proteins are symbolized by nodes, and the connecting edges intricately depict connections between different protein entities.

### 2.2 Protein selection

The network of protein-protein interactions encompasses both virus and human proteins that interact with one another. To identify a suitable drug target, we analyzed the functional connections of human proteins that directly interact with virus proteins utilizing Gene Ontology [46], UniProt [47], and KEGG pathway [48] annotations. In this investigation, we have selected a viral protein as a drug target due to its significant involvement in host-virus interaction, antiviral defense, immunity, mRNA processing, and transcription.

### 2.3 Compound library generation

A library of 537 compounds sourced from *Azadirachta indica* (AI) was selected for this study. The structures and other relevant details of the compounds were acquired from PubChem (https://pubchem.ncbi.nlm.nih.gov/), a public molecular information repository by the National Institutes of Health Roadmap Initiative [49,50].

### 2.4 Validation of protein model quality

An X-ray diffraction structure of the capsid protein in complex with an inhibitor (PDB ID: 6vg5) was retrieved from the Protein Data Bank, demonstrating a resolution of 1.50 Å [21]. To facilitate a thorough quality analysis and validation of the protein model, we employed the ProSA web server [51] and the MolProbity validation program [52]. ProSA evaluates the overall quality of the protein structure for each chain [53], while MolProbity assesses the model's quality on both global and local scales [54]. For MolProbity validation, it was necessary to add missing hydrogen atoms to the model (PDB ID: 6vg5). The hydrogen addition process was implemented using the ‘No flips' method, which incorporated electron cloud x-H bond lengths and van der Waals radii.

### 2.5 Protein and ligand preparation

The PDB file for the capsid protein and inhibitor complex (PDB code: 6vg5) was processed in Biovia Discovery Studio Client 2021 [55] to separate the protein and the reference inhibitor ST-148. The PDB file was cleaned by removing any water molecules and hetero atoms if present, then only the protein chains without ST-148 was saved as receptor in PDB file format. This was followed by the conversion of both PDB files into PDBQT files, utilizing the macromolecule and ligand preparation tools in the AutoDock Suite [56].

The 3D structures of the compounds and the reference inhibitor ST-148 were generated in MOL2 file format using ChemDraw 3D Pro 12.0 software [57]. This molecular modeling software, provided by PerkinElmer Inc., offers advanced capabilities for accurately depicting molecular geometries and conducting energy minimization. The MM2 force field [58], integrated within ChemDraw 3D, was utilized for energy minimization, taking advantage of its ability to calculate molecular energies and optimize conformations. This approach aimed to stabilize the ligand structures and eliminate any unfavorable conformations, thereby facilitating robust and reliable molecular docking studies. Finally, all ligands were converted into PDBQT files using AutoDock Suite [56]. This thorough ligand preparation process was essential for conducting subsequent analyses of ligand-receptor binding affinities and interaction patterns [54].

### 2.6 Molecular docking

The molecular docking procedure was conducted using AutoDockFR suite 1.0 [59] developed by the Department of Integrative Structural and Computational Biology at The Scripps Research Institute. We followed the stepwise protocol suggested by the developers. The ADFR Graphical User interface (agfrgui) was used to position and size the docking box over the receptor pocket(s) into which a ligand was docked, an affinity map for a given list of AutoDock4 atoms types was calculated by the AutoGrid4 program. *The ADFR score, as described in* Equation (1)*, utilizes this energy function independently assessing the interactions among three categories of atoms: Ligand atoms (L), Rigid Receptor atoms (RR), and Flexible Receptor atoms (FR). The total score is derived from the summation of these interaction terms*.

(Eq 1) $$S_{\mathrm{ADFR}}=E_{L-L}+E_{L-RR}+E_{L-FR}+E_{\mathrm{FR}-\mathrm{FR}}+E_{\mathrm{FR}-\mathrm{RR}}$$

For molecular docking using a rigid receptor model only the first two terms are considered: EL-L (ligand intra-molecular interactions) and EL-RR (inter-molecular interactions between the ligand and the rigid receptor).

The receptor and ligand files were loaded in PDBQT format and the docking box was defined based on the binding pocket for the inhibitor ST-148 with the default padding (4.0 Å) added to each side. The ligand binding pockets were computed using AutoSite 1.0 [60]. The binding pocket with the best AutoSite (AS) score was selected for docking of the compounds. The docking-box was re-centered around the selected binding pocket with the default padding (4.0 Å) added to each side. The docking box parameters are: center - x:19.067, y: 5.447, z: 27.313; size - x:14.250, y: 16.500, z: 19.500; padding: 4.000; spacing: 0.375, smoothing: 0.500. An affinity map file was created for each compound. This protocol produces maps that facilitate the search by providing a gradient for resolving clashes and by removing buried favorable cavities too small to accommodate a ligand e.g., trapped water cavities. Subsequently, the ligand was docked into the selected binding pocket using the map files.

### 2.7 Molecular dynamics simulation

A molecular dynamics simulation (MDS) was performed for 100 ns using the Schrödinger-Desmond module to explore the dynamic characteristics and stability of protein-ligand complexes [61]. The simulation system utilized the OPLS3e force field in a 10 × 10 × 10 Å³ orthorhombic box to mimic the protein's actual environment [62]. Na^+^ and Cl^−^ ions were introduced at a salt concentration of 0.15 M to maintain electrical neutrality. The MDS was executed under the isothermal-isobaric (NPT) ensemble at 300 K and a specified pressure of 1.01325 bar. The trajectory output for the 100 ns simulation was obtained using the OPLS-2005 force field. Various analyses, including root-mean-square fluctuation (RMSF), solvent-accessible surface area (SASA), radius of gyration (Rg), polar surface area (PSA), root-mean-square deviation (RMSD), molecular surface area (MoLSA), and protein-ligand contact maps, were conducted to assess stability and interactions between the capsid protein and the lead compound complexes.

### 2.8 MM-GBSA analysis

The binding free energy (ΔG_bind_) of the primary compounds with the capsid protein was determined utilizing Molecular mechanics with Generalized Born surface area (MM-GBSA) [61]. The Prime MM-GBSA module in the Maestro Schrödinger package (Release 2020–3) was employed with default settings to perform this calculation.

### 2.9 Lipinski's drug-likeliness analysis

The drug-like physical and chemical properties of the top-scored compounds were estimated using SwissADME web tool [63].

### 2.10 Pharmacokinetic and toxicological profile analysis

The pkCSM software [64] to assess the absorption, distribution, metabolism, excretion and toxicological profiles of the top-scored compounds.

### 2.11 Human protein target analysis

Human protein targets of the hit compounds were predicted using SwissTargetPrediction, which has a reference set of 224,412 molecules active on 2092 human protein targets. Based on chemical and shape similarity SwissTargetPrediction provides most probable human protein targets of small molecules [65].

## 3 Results and discussion

### 3.1 Findings

#### 3.1.1 Protein-protein interaction network analysis and protein selection

**Fig. 1:**
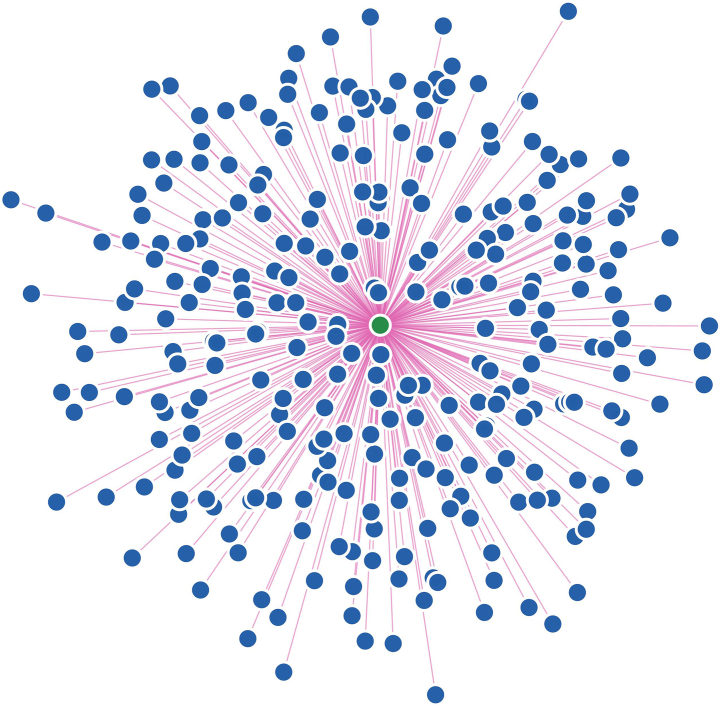
Dengue capsid and human protein-protein interaction network. The green and blue nodes represent capsid and human proteins, respectively.

The dengue virus capsid and human protein interactions network revealed that 264 human proteins (Fig. 1) have direct interactions with capsid protein, and the interactions are distributed in the extracellular region, cytoplasm, and inside the nucleus as well. Molecular functions and biological processes of capsid interacting human proteins involve host-virus interactions, adaptive and innate immunity, antiviral defence, RNA transcription and others. The details of capsid interacting human proteins from the protein-protein interaction network are given as Supplementary Information (Table S1). Some of the important biological processes and capsid interacting human proteins are listed in Table 1.

**Table 1:** Some important dengue virus and human host protein-protein interactions and related biological processes.

Biological process	Human proteins (gene names)
Host-virus interaction	*MOV-10, HNRNPK, DAXX, EXOC1, CCNT1, LCK, HLA-B, UBE2I, TP73, CAMLG, HMGB1, KAT2B, APOE, HLAB, DERL2, FMR1, KPNA1, HSPA5, ANP32B, WWP1, IRAK1*
Antiviral defense	*ZCCHC3, Mx1*
Innate immunity	*ZCCHC3, TRIM26, Mx1, HLA-B, HMGB1, S100A9, HLAB, IRAK4, IRAK1*
Adaptive immunity	*HLA-B, TNF, ZAP70, HMGB1, CD3G, HLAB, CD3E*
mRNA processing	*RRP1B, HNRNPK, RRP12*
Transcription	*RRP1B, MOV-10, HNRNPK, DAXX, EXOC1, CCNT1, TP73, KAT2B*

**Table 2:** Summary statistics of MolProbity structure validation analysis output for the X-ray crystallographic structure of the capsid protein and inhibitor complex (PDB code: 6vg5).

Category	Validation	Raw count	Percentage	Goal
All-Atom Contacts	Clashscore, all atoms	6.02	88^th^ percentile^α^ (N = 598, 1.50 Å ± 0.25 Å)	
Protein Geometry	Poor rotamers	1	0.71 %	Goal: <0.3 %
	Favored rotamers	137	97.16 %	Goal: >98 %
	Ramachandran outliers	0	0.00 %	Goal: <0.05 %
	Ramachandran favored	155	98.10 %	Goal: > 98 %
	Rama distribution Z-score		1.81 ± 0.61	Goal: abs(Z score) < 2
	MolProbity score	1.33	94^th^ percentile^β^ (N = 4836, 1.50 Å ± 0.25 Å)	
	Cβ deviations >0.25 Å	0	0.00 %	Goal: 0
	Bad bonds	38/1466	2.59 %	Goal: 0 %
	Bad angles	0/1949	0.00 %	Goal: <0.1
Peptide Omegas	Cis Prolines	0/6	0.00 %	Expected: ≤1 per chain, or ≤5 %
Additional validation	Chiral volume outliers	0/206		
	Waters with clashes	5/54	9.26 %	

^α^The 100^th^ percentile represents the top performance among structures with similar resolution, while the 0^th^ percentile indicates the lowest performance. ^β^The MolProbity score integrates the clashscore, rotamer, and Ramachandran assessments into one score, adjusted to align with the X-ray resolution scale.

#### 3.1.2 Validation of protein structure quality

**Fig. 2:**
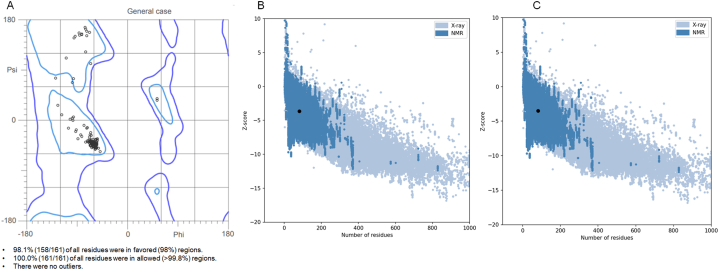
Quality analysis of the X-ray crystallographic model of the capsid protein and inhibitor complex. In this figure, (A) shows Ramachandran plot, (B) and (C) represent overall model quality of Chain A and Chain B of the model, respectively.

Table 2 provides a detailed overview of the protein quality assessments, which includes an all-atom contact analysis and geometric validation performed by MolProbity. Additionally, Fig. 2 depicts the overall model quality evaluated by ProSA-web assessments. The Ramachandran plot (Fig. 2A) for assessment of the overall quality of the X-ray crystallographic structure of the capsid protein and inhibitor complex (PDB code: 6vg5) indicated 98.1 % of the amino acid residues are in favored regions, 100 % of the amino acid residues are in allowed regions, and there were no outliers [66] (Table 2). The Z-score of the protein model −3.64 for Chain A (Fig. 2B) and −3.51 for Chain B (Fig. 2C) indicated the good quality of the structure, based on the X-ray and NMR calculations [67].

#### 3.1.3 Binding affinities and interactions of hits

The ADFR re-docking of the reference inhibitor ST-148 with the capsid protein resulted in a binding affinity of −8.5 kcal/mol, establishing this value as the cutoff criterion for hit selection in our screening process [68]. In the AI compound library, 81 compounds out of 537 demonstrated a binding affinity equal to or exceeding −8.5 kcal/mol with the capsid protein (Tables 3 and S2).

**Table 3:** Binding affinity and number of different types of interactions of the compounds with higher binding energy than that of ST-148.

Compound	Binding energy (kcal/mol)	Number of H-bond(s)	Number of hydrophobic interaction(s)	Number of unfavorable interaction(s)
ST-148	−8.5	2	10	2
AI-6	−9.6	1	0	0
AI-24	−9.4	0	4	0
AI-25	−9.7	0	8	0
AI-27	−9.0	2	0	0
AI-30	−8.8	0	5	0
AI-35	−8.6	0	1	0
AI-36	−8.9	1	0	1
AI-37	−8.8	1	0	1
AI-40	−8.7	1	2	0
AI-45	−8.6	1	2	1
AI-47	−8.9	1	0	0
AI-59	−9.5	5	1	0
AI-61	−9.1	0	7	0
AI-62	−8.6	2	0	2
AI-64	−8.6	1	1	0
AI-65	−9.1	2	0	0
AI-71	−8.9	0	1	0
AI-72	−8.6	0	2	0
AI-75	−9.1	3	0	0
AI-76	−8.5	2	0	0
AI-78	−8.8	0	1	0
AI-79	−10.0	1	1	0
AI-82	−9.0	2	1	0
AI-84	−10.2	1	2	0
AI-87	−10.1	2	2	0
AI-88	−8.6	0	8	0
AI-91	−9.1	2	1	0
AI-93	−8.5	1	0	0
AI-94	−9.1	2	1	0
AI-96	−8.8	1	8	0
AI-103	−9.4	2	1	0
AI-108	−10.3	2	2	0
AI-109	−8.5	0	7	0
AI-110	−8.9	1	1	1
AI-111	−9.7	2	1	0
AI-115	−9.0	4	0	0
AI-116	−9.3	1	1	0
AI-117	−9.3	1	1	1
AI-118	−9.5	0	4	0
AI-121	−8.7	1	0	0
AI-128	−9.4	1	1	0
AI-130	−10.0	1	0	0
AI-131	−9.0	2	0	0
AI-133	−8.8	3	0	0
AI-136	−8.5	2	0	0
AI-137	−9.7	0	3	0
AI-138	−9.4	3	0	0
AI-140	−9.0	3	1	0
AI-142	−9.3	1	6	0
AI-149	−9.9	2	0	0
AI-150	−9.0	1	0	0
AI-153	−10.0	4	1	0
AI-154	−8.5	2	3	0
AI-157	−9.3	1	0	0
AI-158	−10.2	1	2	0
AI-159	−9.5	1	2	0
AI-160	−9.5	1	2	0
AI-161	−9.4	2	0	0
AI-162	−9.9	1	2	0
AI-163	−10.0	1	2	0
AI-164	−9.0	2	0	0
AI-165	−8.7	2	0	0
AI-166	−8.8	1	3	0
AI-169	−9.3	1	3	0
AI-175	−8.9	1	0	0
AI-176	−8.5	3	0	0
AI-178	−8.8	2	0	0
AI-179	−9.8	1	1	0
AI-180	−9.2	1	0	0
AI-181	−10.4	3	1	0
AI-185	−9.3	2	5	0
AI-186	−9.6	1	1	0
AI-187	−9.6	2	1	0
AI-188	−9.0	0	3	0
AI-189	−8.9	1	4	0
AI-190	−8.9	2	1	0
AI-193	−9.5	1	1	0
AI-198	−9.5	2	1	0
AI-199	−10.2	1	2	0
AI-202	−10.1	1	2	0
AI-203	−9.2	2	0	0

#### 3.1.4 Binding modes of top-scored hits

In our analysis, we assessed the binding affinity and modes of interaction, encompassing H-bonds (HB), hydrophobic interactions, and other favorable and unfavorable interactions. We selected 81 hit compounds with a binding affinity equal to or higher than the inhibitor, ST-148. Then, we prioritized the compounds with the highest binding energy and the most hydrogen bonds while ensuring no unfavorable interactions during the selection process (Table 3). Among these, AI-59, also known as epoxy nimonol, exhibited the highest number of H-bonds with a binding affinity score of −9.5 kcal/mol. Additionally, compound AI-181, identified by the chemical name [(5S,7R,8R,9R,10R,13R,17R)-17-[(2R)-2-hydroxy-5-oxo-2H-furan-4-yl]-4,4,8,10,13-pentamethyl-3-oxo-5,6,7,9,11,12,16,17-octahydrocyclopenta[a]phenanthren-7-yl]acetate, displayed the highest binding affinity score of −10.4 kcal/mol.

**Fig. 3:**
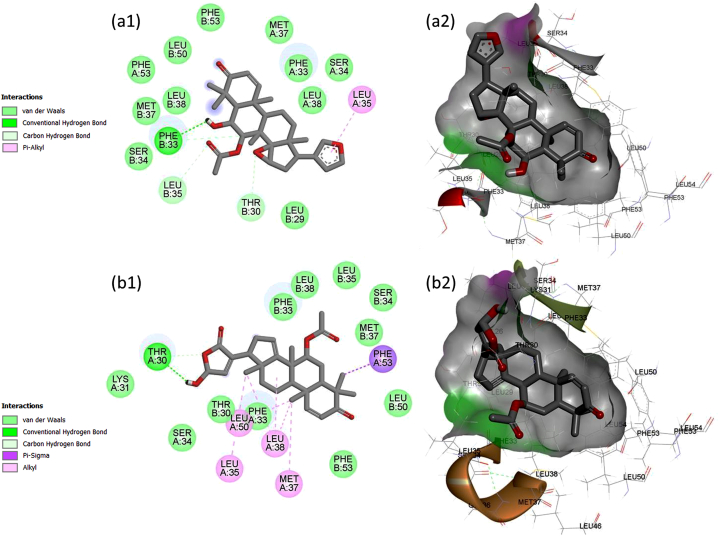
Binding modes of the top-scored hits demonstrating the key interacting residues. Each color represents a unique type of interaction that plays a vital role in the formation of complexes with the capsid protein. Figure (A) and (B) represents the binding interaction pattern of AI-59, and AI-181, respectively.

The following discussion pertains to the binding modes of both compounds. As illustrated in Fig. 3a, epoxy nimonol (AI-59) occupies the ST-148 pocket in capsid protein surrounding the residues A:Thr30, A:Phe33, A:Ser34, A:Leu35, A:Met37, A:Leu38, A:Leu50, A:Phe53, B:Leu29, B:Thr30, B:Phe33, B:Ser34, B:Leu35, B:Met37, B:Leu38, B:Leu50, B:Phe53, B:Leu54. The compound makes five H-bonds: a conventional HB and a carbon HB by donating hydrogen to B:PHE33:O (2.12 Å, 2.87 Å), and three carbon HBs by accepting hydrogen from B:THR30 (2.55 Å, 2.90 Å) and B:LEU35 (2.67 Å) (Table 4). In the vicinity of the furan ring, a pi-alkyl hydrophobic interaction with A:LEU35 (4.34 Å) is established through the pi-orbitals. The remaining residues interact through van der Waals forces (Fig. 3a).

**Table 4:** Summary of interactions between the top-scored hits and inhibitor ST-148 docked against capsid protein.

Compound	Mode of interactions					
	Types	Distance (Å)	From	From Chemistry	To	To Chemistry
AI-59	Con.H-bond	2.13	Ligand:H	H-Donor	B:PHE33:O	H-Acceptor
	CH-Bond	2.56	B:THR30:HA	H-Donor	Ligand:O	H-Acceptor
	CH-Bond	2.90	B:THR30:HB	H-Donor	Ligand:O	H-Acceptor
	CH-Bond	2.67	B:LEU35:HA	H-Donor	Ligand:O	H-Acceptor
	CH-Bond	2.87	Ligand:C	H-Donor	B:PHE33:O	H-Acceptor
	Pi-Alkyl	4.35	Ligand	Pi-Orbitals	A:LEU35	Alkyl
AI-181	Con.H-Bond	1.78	Ligand:H	H-Donor	A:THR30:O	H-Acceptor
	CH-Bond	2.79	A:THR30:HA	H-Donor	Ligand:O	H-Acceptor
	CH-Bond	2.24	A:THR30:HB	H-Donor	Ligand:O	H-Acceptor
	Pi-Sigma	3.49	Ligand:C	C-H	A:PHE53	Pi-Orbitals
ST-148	CH-Bond	2.86	A:THR30:HB	H-Donor	Ligand:N	H-Acceptor
	H-Bond	2.93	A:LEU35:HN	H-Donor	Ligand	Pi-Orbitals
	Pi-Sulfur	5.51	Ligand:S	Sulfur	A:PHE33	Pi-Orbitals
	Pi-Lone Pair	2.82	A:PHE33:O	Lone Pair	Ligand	Pi-Orbitals
	Pi-Lone Pair	2.59	A:PHE33:O	Lone Pair	Ligand	Pi-Orbitals
	Alkyl	5.32	B:LEU38	Alkyl	Ligand	Alkyl
	Alkyl	4.67	B:LEU50	Alkyl	Ligand	Alkyl
	Alkyl	5.07	B:LEU50	Alkyl	Ligand	Alkyl
	Pi-Alkyl	4.02	A:PHE33	Pi-Orbitals	Ligand	Alkyl
	Pi-Alkyl	4.71	B:PHE53	Pi-Orbitals	Ligand	Alkyl
	Pi-Alkyl	3.39	B:PHE53	Pi-Orbitals	Ligand	Alkyl
	Pi-Alkyl	4.13	Ligand	Pi-Orbitals	A:LEU35	Alkyl
	Unfavorable	2.31	A:PHE53	Steric	Ligand:C	Steric
	Unfavorable	2.94	A:THR30:O	H-Acceptor	Ligand:N	H-Acceptor

Con.H-bond = Conventional hydrogen bond, CH-Bond = Carbon hydrogen bond, H-Bond = Hydrogen bond

The binding pocket of AI-181 includes 23 residues from both chains, whereas Thr30, Lys31, Phe33, Ser34, Leu35, Met37, Leu38, Leu50, Phe53, and Leu54 are from chain A and Val26, Leu29, Thr30, Phe33, Ser34, Leu35, Gly36, Met37, Leu38, Leu46, Leu50, Phe53, and Leu54 are from Chain B (Fig. 3b). The 5-hydroxy furan-2(3H)-one ring system in the compound enables the formation of three hydrogen bonds, including one conventional HB (1.78 Å) and two carbon HB (2.78 Å and 2.24 Å) with THR30 in Chain A. The hydroxyl group at the 5-position of the compound serves as the hydrogen bond donor, and the oxygen atom in the 5-position functions as the hydrogen bond acceptor. The hydrophobic interactions consist of one pi-sigma interaction with A:PHE53 and six alkyl interactions with A:LEU38, A:LEU35, A:LEU38, A:MET37, A:LEU38, and A:LEU50 (Table 4). Other residues in the binding pocket, such as B:THR30, B:PHE53, B:LEU50, B:MET37, B:SER34, B:LEU35, B:LEU38, and B:PHE33, are also drawn to the compound through van der Waals forces (Fig. 3b).

The re-docking assessment of ST-148 within the capsid protein structure (PDB ID: 6vg5) is outlined in Table 4. The binding mode analysis indicates that ST-148 establishes favorable hydrogen bonding and hydrophobic interactions, albeit accompanied by two unfavorable contacts. In comparison to ST-148, the compounds AI-59 and AI-181 demonstrated a favorable interaction profile, exhibiting no unfavorable contacts throughout the docking evaluation (Table 4).

#### 3.1.5 Molecular dynamics simulation of top-scored hits

In drug development, molecular dynamics (MD) simulation is a computational technique utilized to assess the stability of protein-ligand complexes by simulating their interactions over time. This approach provides valuable insights into the dynamic behaviour and evolution of the system to understand the complex molecular structures and their interactions [69,70]. In our analysis, we used the Desmond tool to examine trajectory outputs, focusing on key metrics such as radius of gyration (Rg), root mean square fluctuation (RMSF), solvent accessible surface area (SASA), polar surface areas (PSA), root-mean-square deviation (RMSD), molecular surface area (MoLSA), and protein-ligand contact.

**Fig. 4:**
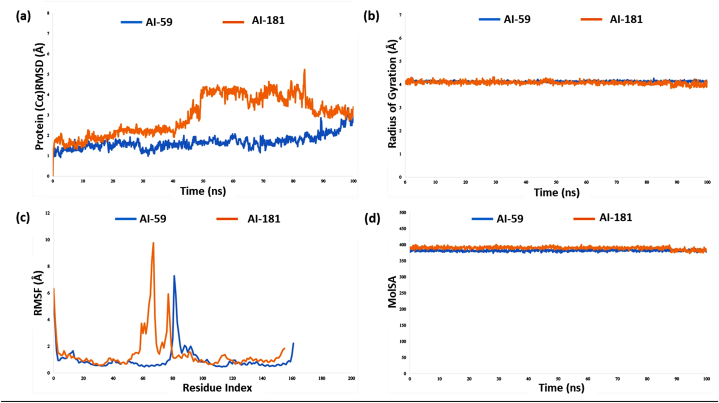
The root-mean-square deviation, radius of gyration, root mean square fluctuation and molecular surface area values of a 100 ns molecular dynamics simulation study. In this figure, (A) shows the RMSD values of capsid protein and compound AI-59 (blue) and AI-181 (orange) complexes; (B) shows the Rg values, (C) shows the RMSF values of the Cα atoms in the protein-ligand complexes, and (D) shows the MoLSA values.

Root-mean-square deviation (RMSD) is extensively employed in computer-aided drug development to measure the conformational stability of protein-ligand complexes. An acceptable RMSD range for protein-ligand complexes is typically 1–4 Å. Values outside this range reflect severe conformational changes in the protein structure [61]. For analysis the structural stability of the protein, the RMSD values of capsid and hit compound complexes were computed over 100 ns (Fig. 4a). Here, the capsid and AI-59 complex showed minimal RMSD value of 0.921 Å, which were below 2 Å up to 88 ns. However, at last the RMSD value reached the maximum of 3.04 Å. On the other hand, AI-181 showed minimal fluctuations from 1.23 to 2.29 Å until 40 ns. Then it showed a sharp increase and irregular fluctuation up to 4.83 Å at 78 ns. The data indicate the stability of the two compounds, with average RMSD values ranging from 1.1 to 3.1 Å, suggesting minimal variance. Throughout the experimental periods, AI-59 was more stable than AI-181.

The variation in stability and affinity between the top-scored compounds, AI-59 and AI-181 may be explained by their binding modes especially H-bond characteristics. In binding mode analysis it was observed that the compound AI-59 could stabilize the complex through 5 H-bonds to the capsid protein (Table 1). Whereas the compound AI-181 could make 3 H-bonds to the protein. Interestingly, the compound AI-181 has higher binding affinity to the capsid protein and made a conventional HB with a distance value of 1.78 Å (Table 3), which is lower than the conventional HB by the compound AI-59 (2.13 Å). Hydrogen bonding (HB) is critical in directing and recognizing substrates by modifying their affinity for binding partners. HB network in the binding site maintains structure and function with high-level connectivity. The combined influence of the type, quantity, and distance of hydrogen bonds between donors and acceptors is essential in determining affinity and stability [71].

The radius of gyration (Rg) quantifies the spatial distribution of atoms in relation to the principal axis of a protein-ligand complex, providing insight into the structural compactness of the complex [72]. We monitored the Rg values for the complexes containing capsid protein and the top-scored compounds AI-59 and AI-181 for 100 ns (Fig. 4b). The average Rg values of the compounds AI-59 and AI-181 were 4.0 Å and 4.1 Å, respectively. The compound AI-181 showed a fluctuation of Rg values between 3.924 Å and 4.322 Å, and AI-59 showed a fluctuation between 4.268 Å and 4.036 Å. The complexes did not demonstrate any substantial structural changes following the binding process.

RMSF is utilized for monitoring local changes in a protein by detecting the displacement of individual atoms in comparison to a reference structure [73]. Fig. 4c displays the RMSF for the residue Cα of the capsid protein and inhibitor complex with the compound AI-59 (blue) and AI-181 (orange). The RMSF analysis revealed significant fluctuations between residues 60-80 and 80-88 for AI-181 and AI-59, with maximum ranges of 0.7 nm and 0.4 nm, respectively, indicating low stability at these positions. Moreover, both compounds exhibited considerable stability for the remaining positions. The results showed that AI-59 performed better than AI-181 in terms of Rg.

MoLSA, equivalent to the van der Waals surface area with a 1.4 probe diameter, was utilized to evaluate the chosen compounds [74]. Fig. 4d shows the MoLSA values for the compounds AI-59 and AI-181. The MoLSA value for AI-59 ranges from 373.76 Å^2^ to 390.86 Å^2^ and for AI-181 from 372.86 Å^2^ to 400.55 Å^2^.

**Fig. 5:**
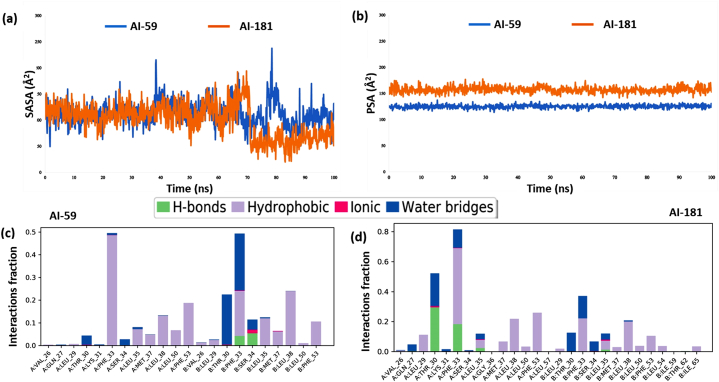
The solvent-accessible surface area, polar surface areas and protein-ligand interaction profiles over a 100 ns molecular dynamics simulation study. In (a), the diagram shows the solvent-accessible surface area (SASA) of protein-ligand interactions. Here, the blue color represents the compound AI-59, and the orange color represents the compound AI-181. (b) shows the polar surface area of the compounds interacting with the capsid protein. The stacked bar charts in (c) and (d) reflect the interactions between capsid protein and the compound AI-59 and AI-181, respectively.

Measuring the solvent-accessible surface area (SASA) is crucial for analyzing the structure and behaviour of complex molecules, particularly in their interactions with drug-like compounds [75]. Fig. 5a displays the SASA values for the capsid protein when bound to AI-59 and AI-181, demonstrating fluctuations in the range of 100 Å^2^ to 200 Å^2^ and 40 Å^2^ to 100 Å^2^, respectively.

Polar surface area is defined as the molecular surface area contributed by polar atoms, specifically nitrogen and oxygen atoms in conjunction with their attached hydrogen atoms [76]. In this case, the top-scored compounds AI-59 (blue) and AI-181 (orange) complexes with the capsid protein have a range of PSA from 120 to 130 Å^2^ and 150 to 160 Å^2^, respectively, with slight fluctuations during the simulation (Fig. 5b). Throughout the simulation, AI-181 consistently maintains a higher PSA value than AI-59.

The interactions between the capsid protein and the top-scored compounds AI-59 and AI-181 were analyzed for 100 ns of simulation. Our analysis encompassed ionic interactions, hydrophobic interactions, water bridges, and hydrogen bonds. Fig. 5c and d visually demonstrate the consistent binding of the ligands to the protein over the entire simulation.

#### 3.1.6 Post simulation binding energy analysis (MM/GBSA) of top-scored hits

**Fig. 6:**
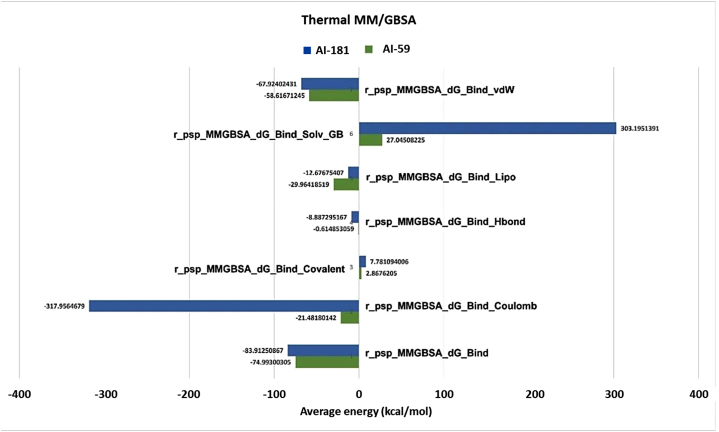
Post-simulation MM/GBSA analysis of the capsid protein and top-scored hits compound complexes.

The MM/GBSA approach, a molecular dynamics (MD) based method, is proficient at estimating binding affinities. This ‘end-state' method evaluates binding energy by analyzing the intermolecular interactions between the protein and ligand using configurations obtained from the MD trajectory [77]. MM/GBSA calculations for the capsid protein complexes with AI-59 and AI-181 produced negative ΔG_bind (NS) scores of −74.99 and −83.91 kcal/mol, respectively. van der Waals interaction energy (ΔG_bind vdW), the relevance of lipophilicity energy (ΔG_bind lipo), and Coulomb energy (ΔG_bind Coulomb) contributed to the stability of the complexes (Fig. 6).

#### 3.1.7 Drug-like physicochemical properties of the top-scored hits

Lipinski's Rule of Five (Ro5) is a commonly used standard for evaluating a compound's suitability as an oral drug [78]. The drug-like properties of the top-scored compounds meet the standards of oral drugs according to the rule of five. In Table 5, we summarized the drug-likeness of the top-scored compounds. We provided physicochemical properties of the top 81 hit compounds and their medicinal chemistry-related properties, Lipinski's rule violations, pan assay interference alerts (PAINS), and synthetic accessibility in the supplementary information (Table S3).

**Table 5:** Drug-like properties of the top-scored hits based on Lipinski's Rule of Five.

	AI-59	AI-181
Molecular weight	468.58 g/mol	468.58 g/mol
Octanol-water partition coefficient (log P)	3.67	3.88
Hydrogen bond acceptors (HBA)	6	6
Hydrogen bond donors (HBD)	1	1
Number of rotatable bonds (RB)	3	3
Topological polar surface area (TPSA)	89.27 Å^2^	89.90 Å^2^

#### 3.1.8 Pharmacokinetic and toxicological profile of the top-scored hits

We used pkCSM software online [64] to determine the oral absorption, distribution, metabolism, excretion, and toxicity (ADMET) of the top-scored compounds, AI-59 and AI-181. The findings outlined in Table 6 show that the top-scored compounds have high Caco-2 permeability, which means the substance can be quickly absorbed [64]. Caco-2 human colon carcinoma cell line's monolayer permeability coefficient is a reliable method to predict the absorption rate of orally administered drugs. Researchers widely use the Caco-2 cell model to evaluate *in vitro* human intestinal permeability due to its similarity to human enterocytes [79].

**Table 6:** Computed ADMET properties of the top-scored hits

Properties	AI-59	AI-181	Unit	Value	Interpretation
Absorption					
Water solubility	−4.652	−5.362	log mol/L		
Caco2 permeability	0.906	0.978	log cm/s	>0.90	Highly permeable
Human intestinal absorption	98.57	97.89	% Absorbed	<30 %	Poorly absorbed
Skin Permeability	−3.297	−3.825	log Kp (cm/h)	>-2.5	Low permeability
P-glycoprotein substrate	No	No	Categorical		Yes/No
P-glycoprotein I inhibitor	Yes	Yes	Categorical		Yes/No
P-glycoprotein II inhibitor	No	Yes	Categorical		Yes/No
Distribution					
VDss	0.166	−0.053	log L/kg	<-0.15	Low VDss
				>0.45	High VDss
Fraction unbound	0.064	0.019	Numeric (Fu)		
BBB permeability	−0.387	−0.337	log BB	>0.3	Readily permeable
				<-1	Poorly permeable
CNS permeability	−2.871	−2.302	log PS	>-2	Permeable
				<-3	Not permeable
Metabolism					
CYP2D6 substrate	No	No	Categorical		Yes/No
CYP3A4 substrate	Yes	Yes	Categorical		Yes/No
CYP1A2 inhibitor	No	No	Categorical		Yes/No
CYP2C19 inhibitor	No	No	Categorical		Yes/No
CYP2C9 inhibitor	No	No	Categorical		Yes/No
CYP2D6 inhibitor	No	No	Categorical		Yes/No
CYP3A4 inhibitor	Yes	No	Categorical		Yes/No
Excretion					
Total Clearance	0.13	0.17	log (ml/min/kg)		
Renal OCT2 substrate	No	Yes	Categorical		Yes/No
Toxicological					
AMES toxicity	No	No	Categorical		Yes/No
Maximum tolerated dose (human)	−0.88	−0.75	log (mg/kg/day)		≤0.477 Low
					>0.477 high
hERG I inhibitor	No	No	Categorical		Yes/No
hERG II inhibitor	No	No	Categorical		Yes/No
Oral rat acute toxicity	2.87	2.16	mol/kg		
Oral rat chronic toxicity	0.52	0.16	log (mg/kg_bw/day)		
Hepatotoxicity	No	No	Categorical		Yes/No
Skin sensitization	No	No	Categorical		Yes/No

Our research revealed that the compounds we investigated demonstrate excellent intestinal absorption, 98.57 % for AI-59 and 97.89 % for AI-181 (Table 6). According to the pkCSM computation method, a substance with human intestinal permeability below 30 % is inadequately absorbed [64]. Notably, neither compound is a substrate for P-glycoprotein, eliminating concerns about efflux from cells but have possibility of drug-drug interactions to drugs that are P-glycoprotein inhibitors [80,81].

The distribution of drugs in the body is crucial for medication development. *In silico* techniques can accurately analyze factors such as steady-state volume of distribution (VDss), blood-brain barrier (BBB) crossing, and central nervous system (CNS) interaction. Determining plasma protein interactions is vital in drug development, as only unbound drugs can interact with protein targets [82]. AI-181 has a lower distribution volume than AI-59. Both compounds cannot cross the blood-brain barrier and lack CNS permeability (Table 6).

Cytochrome enzymes play a crucial role in metabolizing drugs through oxidation. Among these enzymes, CYP3A is the most prevalent in humans and is responsible for processing about 60 % of drugs, steroids, carcinogens, and eicosanoids [83,84]. Our findings indicate that neither of the top-scored compounds are substrates of CYP2D6, but both are substrates of CYP3A4. Furthermore, none of the compounds showed inhibitory effects on CYP1A2, CYP2C19, CYP2C9, and CYP2D6 (Table 6).

Based on the Total Clearance data in Table 6, both compounds demonstrate similar bioavailability, indicating that comparable dosing regimens might be necessary to reach steady-state plasma concentrations [85]. The renal organic cation transporter 2 (OCT2) transportability of drug candidates provides valuable insights into drug clearance and potential contraindications [86,87]. The pkCSM prediction identified AI-181 as the substrate for renal OCT2, while AI-59 is not (Table 6).

In a drug discovery process, it is imperative to assess acute toxicity in mammals, particularly rats or mice, to ensure safety. The median lethal dose (LD_50_) serves as a measure of acute toxicity, indicating the quantity of a tested substance necessary to cause fatality in 50 % of treated animals within a specified timeframe. Chronic toxicity studies, typically over 6–12 months, play a significant role in identifying the lowest observed adverse effect level (LOAEL) and the no-observed-adverse-effect level (NOAEL) for a compound. This data is crucial for understanding the potential clinical risks associated with long-term treatment at the anticipated clinical dose [88,89]. The pkCSM software provides estimated oral rat acute and chronic toxicity data, as depicted in Table 6.

In pharmacology, the maximum tolerated dose (MTD) represents the highest amount of a drug that can be administered to a human subject over a defined period without causing harm to their survival [90]. This value is pivotal in determining the appropriate starting dose for phase I clinical trials of a drug [91]. A drug with an MTD greater than 0.477 log (mg/kg/day) is highly toxic. According to predictions by the pkCSM software, AI-59 and AI-181 are deemed non-toxic [64]. These compounds exhibit no mutagenicity, hepatotoxicity, or skin sensitization (Table 6).

The hERG (human Ether-à-go-go-Related Gene) potassium channels I and II are crucial in regulating the heart's electrical activity. Certain drugs have the potential to be fatal by blocking this channel. Thus, it is essential to thoroughly evaluate drug candidates for hERG inhibition during the discovery process [92,93]. Our research has shown that none of the top-scored compounds demonstrate hERG inhibitory potential (Table 6) and do not pose a significant risk for cardiotoxicity.

#### 3.1.9 Possible human protein target of top-scored hits

SwissTargetPrediction analysis determined that none of the compounds (AI-59 and AI-181) have probable human protein targets with a probability score exceeding 0.5. The results are given as supplementary information (Table S4).

### 3.2 Implications

This study utilized a network pharmacology approach to perform a retrospective analysis of dengue virus proteins, representing a novel strategy in drug discovery. Our protein-protein interaction study has conclusively demonstrated that the dengue capsid protein engages with several human proteins that are critically linked to virus-host interactions and antiviral defense, particularly through innate and adaptive immunity mechanisms (Table 1). Based on this compelling data, we assert that inhibiting the capsid protein will significantly impair virus-host interactions, thereby reducing the spread of infections throughout the body. Furthermore, capsid inhibitors hold the potential to bolster the immune response against the virus. A pivotal study by Faustino et al. on the structural and functional properties of the dengue capsid protein, in comparison to other related flaviviruses, supports our findings and indicates that the capsid protein may function as an immunosuppressor, drawing parallels to influenza NS1. The similarities between DENV C and influenza NS1 extend to their RNA-binding capabilities, with the organization of the RNA-binding domain of NS1 from influenza A resembling that of the DENV C protein [94].

The discovery and development of antivirals targeting flaviviruses have posed challenges, prompting researchers to explore non-traditional targeting strategies in the wake of failed conventional approaches such as nucleoside analogs [95], protease inhibitors [96], and entry-directed compounds [97]. This *in silico* methodology for discovering antiviral agents is can be adapted to target the capsid proteins of diverse flavivirus species, which represent significant global health challenges.

In this investigation, we identified potent inhibitors of the DENV-2 capsid protein through a comprehensive screening of 537 compounds from *A. indica*. Our rigorous evaluation process, which included molecular docking and molecular dynamics simulations, demonstrated strong binding affinities of the compounds to the capsid protein (Table 3, Table 4; Fig. 3, Fig. 4, Fig. 5). The findings from the binding mode analysis offer valuable insights for structure-based drug design aimed at the C protein of DENV and other related flaviviruses. The physicochemical and drug-like properties of our compounds (Table 5), as evaluated by Lipinski's Rule of Five, confirm they can be formulated in oral dosage forms [78]. Computed ADMET properties (Table 6) demonstrated that both lead compounds have good absorption from gastrointestinal tract, high volume of distribution with no CNS permeability. Both compounds can possibly be metabolized by CYP3A4 and are not contraindicated to the drugs that are metabolized by CYP1A2, CYP2C19, CYP2C9, CYP2D6 enzymes (Table 6). Furthermore, the pkCSM Toxicological predictions demonstrated no genetic toxicity, cardiotoxicity, hepatotoxicity and skin sensitization. However, both compounds have low maximum tolerated doses in human (Table 6).

Our research aims to develop small-molecule inhibitors targeting the virus protein DENV-2 capsid. The findings from SwissTargetPrediction analysis indicate that these compounds are unlikely to interact with unintended targets within the human body, effectively reducing the risk of off-target toxicities, which often lead to side effects and challenges in clinical trials [98].

### 3.3 Limitations

In this research, we conducted thorough analyses including molecular docking, molecular dynamics simulations, and ADMET predictions of *Azadirachta indica* phytochemicals. However, the lead compounds are still at the preclinical stage, requiring further validation in experimental and clinical settings. *In vitro* cell line assays and *in vivo* animal studies are important for confirming the efficacy of the compounds in inhibiting the function of the capsid protein. Furthermore, it is essential to engage in detailed research to better understand the implications of capsid protein inhibition on virus-host interactions. Gaining insights into these interactions is crucial for evaluating the potential impact of capsid protein inhibition on the overall immune response of the host.

The dengue virus comprises four distinct serotypes, which raises important questions regarding the applicability of findings related to the DENV-2 capsid protein to other viral strains. The C proteins of different flaviviruses are characterized by substantial sequence and structural similarities, exhibiting 55 % sequence conservation among mosquito-borne flaviviruses and an impressive 80 % conservation among different serotypes of dengue Virus (DENV). A comprehensive analysis of flavivirus C proteins, examining the relationship between protein size, thermal stability, and functional dynamics, supports a unified mechanism that underlies their biological activity [94]. Accordingly, the hit compounds identified in this study are highly promising for further studies targeting C protein of other serotypes. Ongoing research is concentrating on the capsid proteins of DENV-1, DENV-3, and DENV-4 serotypes, utilizing a compound library comprising a wide array of bioactive molecules.

## 4 Conclusion

The quest for finding molecules that can block specific proteins has become extremely important in drug development because of its effectiveness and speed. The study presents potential DENV-2 capsid protein inhibitors interacting within the binding pocket of ST-148. This structural protein plays a critical role in virus-host interaction and host immunity. ST-148, an inhibitor of the capsid protein, forms a tetramer of the protein and reduces the infectivity of the progeny virus [21,99]. Our report presents highly promising compounds from *A. indica*, their binding modes, and thorough insights into their pharmacokinetic and toxicological properties. Pharmacokinetic and toxicology investigations indicate that these compounds could be promising candidates for preclinical evaluation [100]. The results of this research can pave the way for the development of DENV-2 capsid protein inhibitors for the treatment of dengue infection.

## CRediT authorship contribution statement

**Md. Ahad Ali Khan:** Writing – review & editing, Writing – original draft, Visualization, Supervision, Methodology, Investigation, Conceptualization. **Md. Nazmul Hasan Zilani:** Writing – original draft, Visualization, Investigation. **Mahedi Hasan:** Writing – original draft, Investigation. **Nahid Hasan:** Data curation.

## Data availability statement

Data included in the article/supplementary material is referenced in the article.

## Declaration of generative AI and AI-assisted technologies in the writing process

In the course of developing this manuscript, the author(s) employed Grammarly for initial grammar verification and corrections. Following this preliminary assessment, the author(s) conducted a comprehensive review and revision of the content, retaining full accountability for the integrity and accuracy of the published work.

## Declaration of competing interest

The authors declare that they have no known competing financial interests or personal relationships that could have appeared to influence the work reported in this paper.

## List of abbreviations

ADMET: Absorption, Distribution, Metabolism, Excretion, and Toxicity
AI: *Azadirachta Indica*
BBB: Blood Brain Barrier
Caco-2: Human Colon Carcinoma Cell Line
CNS: Central Nervous System
CYP: Cytochrome Enzyme
DENV: Dengue Virus
DENV C: Dengue Virus Capsid Protein
DHF: Dengue Hemorrhagic Fever
DNA: Deoxyribonucleic Acid
HB: Hydrogen Bond
HBA: Hydrogen Bond Acceptors
HBD: Hydrogen Bond Donors
hERG: Human Ether-à-go-go-Related Gene
HTS: High-Throughput Screening
KEGG: Kyoto Encyclopedia of Genes and Genomes
LD50: Median Lethal Dose
LOAEL: Lowest Observed Adverse Effect Level
Log P: Octanol-Water Partition Coefficient
MD: Molecular Dynamics
MDS: Molecular Dynamics Simulation
MM-GBSA: Molecular Mechanics, General Born Surface Area
MoLSA: Molecular Surface Area
mRNA: Messenger RNA
MTD: Maximum Tolerated Dose
MW: Molecular Weight
NMR: Nuclear Magnetic Resonance
NOAEL: No-Observed-Adverse-Effect Level
OCT2: Organic Cation Transporter 2
PAINS: Pan Assay Interference Alerts
PDB: Protein Data Bank
PPI: Protein-Protein Interaction
PSA: Polar Surface Area
RB: Number of Rotatable Bonds
Rg: Radius of Gyration
RMSD: Root-Mean-Square Deviation
RMSF: Root-Mean-Square Fluctuation
RNA: Ribonucleic Acid
SASA: Solvent-Accessible Surface Area
TPSA: Topological Polar Surface Area
Vdss: Steady-State Volume of Distribution
vdW: Van Der Waals
