# Supplementary material for: Identification and evaluation of bioactive compounds from Azadirachta indica as potential inhibitors of DENV-2 capsid protein: An integrative study utilizing network pharmacology, molecular docking, molecular dynamics simulations, and machine learning techniques
Source: Heliyon. 2025 Feb 12;11(4):e42594. doi: 10.1016/j.heliyon.2025.e42594 (PMC11883367; doi:10.1016/j.heliyon.2025.e42594)
Supplement: Multimedia component 1 [file mmc1.docx]

**Supplementary Material**

**Title: Identification and Evaluation of Bioactive Compounds from *Azadirachta indica* as Potential Inhibitors of DENV-2 Capsid Protein: An Integrative Study Utilizing Network Pharmacology, Molecular Docking, Molecular Dynamics Simulations, and Machine Learning Techniques.**

**Md. Ahad Ali Khan^a*^, Md. Nazmul Hasan Zilani^b^, Mahedi Hasan^a^, Nahid Hasan^a^**

^a^Department of Pharmacy, Manarat International University, Dhaka, Bangladesh

**^b^**Department of Pharmacy, Jashore University of Science and Technology, Jashore, Bangladesh.

^*^**Corresponding author**

**Md. Ahad Ali Khan**, Assistant Professor, Department of Pharmacy, Manarat International University.

Ashulia Model Town, Khagan, Ashulia-1341, Dhaka, Bangladesh

Email: [ahadali@manarat.ac.bd](mailto:ahadali@manarat.ac.bd), Orchid ID: 0000-0002-7547-7431

**Table S1. A list of human proteins that interact with the dengue virus capsid protein, identified through protein-protein interaction networks between the dengue virus and its human host.**

| **Human protein** | **Human protein** | **Molecular function** | **Biological process** |
| --- | --- | --- | --- |
| ACAD11 | Acyl-CoA dehydrogenase family member 11 | Acyl-CoA dehydrogenase activity, Protein binding, Oxidoreductase activity, Flavin adenine dinucleotide binding | Lipid metabolic process, Fatty acid metabolic process |
| ACY1 | Aminoacylase-1 | Aminoacylase activity, Hydrolase activity, Protein binding, Metal ion binding | Amino acid metabolic process |
| AFTPH | Aftiphilin | Clathrin binding | Protein transport, Intracellular transport |
| ALDOB | Fructose-bisphosphate aldolase B | Lyase activity, ATPase binding, Molecular adaptor activity, Fructose-1-phosphate aldolase Activity, Fructose binding | Fructose metabolic process, Gluconeogenesis, Glycolytic process, NADH oxidation, Positive regulation of ATP-dependent activity |
| ANKRD12 | Ankyrin repeat domain-containing protein 12 | Protein binding, Cytoskeletal anchor activity, Enzyme binding, Protein phosphatase binding, Spectrin binding, Transmembrane transporter binding, ATPase binding | Exocytosis, Endoplasmic reticulum to Golgi vesicle-mediated transport, Cytoskeleton organization, Positive regulation of organelle organization, Protein localization to plasma membrane |
| ANP32B | Acidic leucine-rich nuclear phosphoprotein 32 family member B | Chaperone, Protein binding, Histone binding, RNA polymerase binding | Regulation of apoptotic process, Negative regulation of cell differentiation, Positive regulation of protein export from nucleus, Host-virus interaction |
| ANR12 | Ankyrin repeat domain-containing protein 12 |  |  |
| AP3B1 | AP-3 complex subunit beta-1 | Protein binding, Protein phosphatase binding, GTP-dependent protein binding, Protein phosphatase binding | Cell morphogenesis, Toll-like receptor signaling pathway, Protein transport, Transport |
| APOE | Apolipoprotein E | Heparin-binding | Cholesterol metabolism, Host-virus interaction, Lipid metabolism, Lipid transport, Steroid metabolism |
| ARHG4 | Rho guanine nucleotide exchange factor 4 | Protein binding, Guanine-nucleotide releasing factor | Lamellipodium assembly, Intracellular signal transduction, Filopodium assembly, Regulation of small GTPase mediated Signal transduction |
| BAZ1A | Bromodomain adjacent to zinc finger domain protein 1A | DNA binding, Protein binding, Metal ion binding, Histone acetyltransferase activity | Regulation of DNA replication, Nucleosome assembly, Chromatin remodeling, Transcription,  Transcription regulation |
| BAZ1B | Tyrosine-protein kinase BAZ1B | Zinc ion binding, ATP binding, Protein binding, Transferase activity, Histone kinase activity, Tyrosine-protein kinase | Regulation of transcription by RNA polymerase II, Chromatin organization, DNA damage response, Transcription regulation, Phosphorylation |
| BIRC2 | Baculoviral IAP repeat-containing protein 2 | Transcription coactivator activity, Ubiquitin-protein transferase activity, Protein binding, Transferase activity | Apoptosis process, Transcription, Transcription regulation, Ubl conjugation pathway, Regulation of innate immune response |
| BOD1L | Biorientation of chromosomes in cell division protein 1-like 1 | Protein phosphatase 2A binding, Protein phosphatase inhibitor activity | DNA damage, DNA repair |
| BRX1 | Homeobox protein BarH-like 1 | DNA-binding transcription factor activity,  RNA polymerase II-specific | Regulation of transcription by RNA polymerase II |
| BYSL | Bystin | RNA-binding, Protein binding | Ribosome biogenesis, Regulation of protein localization to nucleolus, rRNA processing |
| CAMLG | Guided entry of tail-anchored proteins factor CAMLG | Protein binding, Ubiquitin protein ligase binding | ER-Golgi transport, Host-virus interaction, Transport, Defense response |
| CC137 | Coiled-coil domain-containing protein 137 | RNA binding, Protein binding |  |
| CCNK | Cyclin-K | Cyclin-dependent protein Serine/threonine kinase activity, Protein binding | DNA damage response, Host-virus interaction, Transcription regulation, Regulation of cyclin-dependent protein serine/threonine kinase activity |
| CCNT1 | Cyclin-T1 | DNA binding, Chromatin binding,  protein binding, Cyclin-dependent protein serine/threonine kinase activity, RNA polymerase binding | Cell cycle, Cell division, Host-virus interaction, Transcription, Transcription regulation, Regulation of cyclin-dependent protein serine/threonine kinase activity |
| CD38 | ADP-ribosyl cyclase/cyclic ADP-ribose hydrolase 1 | Hydrolase activity, Receptor, Transferase activity | B cell proliferation, Female pregnancy, Apoptotic signaling pathway |
| CD3E | T-cell surface glycoprotein CD3 epsilon chain | Transmembrane signaling receptor activity,  T cell receptor binding | Adaptive immunity, Immunity, T cell activation, T cell proliferation, Regulation of apoptotic process, Lymphocyte activation |
| CD3G | T-cell surface glycoprotein CD3 gamma chain | Transmembrane signaling receptor activity, T cell receptor binding | Adaptive immunity, Immunity, Protein transport, T cell activation, Regulation of lymphocyte apoptotic process |
| CDC5L | Cell division cycle 5-like protein | DNA-binding, RNA-binding | RNA polymerase II transcription regulatory region sequence-specific DNA binding, mRNA splicing, via spliceosome, DNA repair, Regulation of transcription by RNA polymerase II, mRNA processing |
| CDK9 | Cyclin-dependent kinase 9 | Kinase activity, Serine/threonine-protein kinase activity, Transferase activity | DNA damage, DNA repair, Host-virus interaction, Transcription, Transcription regulation |
| CLDN1 | Claudin domain-containing protein 1 | Virus receptor activity | Cell adhesion, Symbiont entry into host cell, Xenobiotic transport across blood-nerve barrier, Response to interleukin-18, Bicellular tight junction assembly |
| CLU | Clustered mitochondria protein homolog | RNA-binding, mRNA binding | Intracellular distribution of mitochondria, Mitochondrion organization |
| CMS1 | Protein CMSS1 | RNA binding, Protein binding |  |
| CORO1A | Coronin | Actin filament binding, Identical protein binding | Calcium ion transport, Leukocyte chemotaxis, T cell proliferation |
| CPSF7 | Cleavage and polyadenylation specificity factor subunit 7 | RNA-binding | mRNA processing |
| CSNK2A1 | Casein kinase II subunit alpha | Kinase activity, Serine/threonine-protein kinase activity, Transferase activity | Apoptosis, Biological rhythms, Cell cycle, Transcription, Transcription regulation, Wnt signaling pathway |
| CUL3 | Cullin-3 | Cyclin binding, Ubiquitin protein ligase activity | Cell cycle, Cell division, Cilium biogenesis/degradation, ER-Golgi transport, Mitosis, Transport, Ubl conjugation pathway |
| DAXX | Death domain-associated protein 6 | Chaperone, Chromatin regulator, Repressor | Apoptosis, Host-virus interaction, Transcription, Transcription regulation |
| DDX18 | ATP-dependent RNA helicase DDX18 | Helicase activity, Hydrolase activity, RNA-binding |  |
| DDX21 | Nucleolar RNA helicase 2 | Helicase activity, Hydrolase activity, RNA-binding | Antiviral defense, Immunity, Innate immunity, rRNA processing, Transcription |
| DDX50 | ATP-dependent RNA helicase DDX50 | Helicase activity, Hydrolase activity, RNA-binding |  |
| DDX55 | ATP-dependent RNA helicase DDX55 | Helicase activity, Hydrolase activity, RNA-binding |  |
| DEN1C | DENN domain-containing protein 1C | Guanyl-nucleotide exchange factor activity | Endocytosis |
| DENND1C | DENN domain-containing protein 1C | Guanine-nucleotide releasing factor | Endocytic recycling, Endocytosis |
| DERL2 | Derlin-2 | Misfolded protein binding, Ubiquitin-specific protease binding | Host-virus interaction, Unfolded protein response |
| DGCR8 | Microprocessor complex subunit DGCR8 | RNA-binding | Primary miRNA processing |
| DHX15 | ATP-dependent RNA helicase DHX15 | Helicase activity, Hydrolase activity, RNA-binding | Immunity, Innate immunity, mRNA processing, mRNA splicing |
| DHX30 | ATP-dependent RNA helicase DHX30 | Helicase activity, Hydrolase activity, RNA-binding | Ribosome biogenesis |
| DHX36 | ATP-dependent DNA/RNA helicase DHX36 | Activator, Developmental protein, DNA-binding, Helicase activity, Hydrolase activity, Repressor activity, RNA-binding | Antiviral defense, Differentiation, Immunity, Innate immunity, Transcription, Transcription regulation, Transport |
| DHX57 | Putative ATP-dependent RNA helicase DHX57 | Helicase activity, Hydrolase activity |  |
| DIM1 | Probable dimethyladenosine transferase | Methyltransferase activity, RNA-binding, Transferase activity | rRNA processing |
| DNTTIP2 | Deoxynucleotidyltransferase terminal-interacting protein 2 | RNA binding | Transcription, Transcription regulation |
| EMAL2 | Echinoderm microtubule-associated protein-like 2 | Protein binding, Microtubule binding, Tubulin binding, Protein self-association | Microtubule cytoskeleton organization, Visual perception, Sensory perception of sound, Regulation of microtubule nucleation, Negative regulation of microtubule polymerization |
| EXOC1 | Exocyst complex component 1 | Phosphatidylinositol-4,5-bisphosphate binding | Antiviral defense, Exocytosis, Host-virus interaction, Protein transport, Transport |
| EXOS3 | Exosome complex component RRP40 | RNA exonuclease activity, 3'-5'-RNA exonuclease activity, RNA binding, Protein binding | rRNA processing, RNA processing,  RNA catabolic process, DNA deamination |
| EXOS7 | Exosome complex component RRP42 | RNA exonuclease activity, 3'-5'-RNA exonuclease activity, RNA binding, protein binding | rRNA processing, RNA processing, RNA catabolic process |
| F120A | Constitutive coactivator of PPAR-gamma-like protein 1 | RNA binding |  |
| FAM192A | PSME3-interacting protein |  | Negative regulation of protein binding |
| FMR1 | Fragile X messenger ribonucleoprotein 1 | Activator, Repressor, Ribonucleoprotein, RNA-binding | DNA damage, Host-virus interaction, mRNA processing, mRNA splicing, mRNA transport, Neurogenesis, RNA-mediated gene silencing, Translation regulation |
| G3BP1 | Ras GTPase-activating protein-binding protein 1 | Nucleic acid binding (DNA & RNA), Helicase activity (DNA & RNA), Nuclease & endonuclease activity, Protein binding, ATP binding, Ribosomal small subunit binding, Hydrolase activity, | Immune system process, Positive regulation of type I interferon production, DNA duplex unwinding, Innate immune response, Defense response to virus, |
| G45IP | Large ribosomal subunit protein mL64 | Protein binding | Cell cycle, Mitochondrial translation |
| GAB1 | GRB2-associated-binding protein 1 | Signaling adaptor activity | Actin cytoskeleton reorganization, Angiogenesis, Epidermal growth factor receptor signaling pathway |
| GLYR1 | Cytokine-like nuclear factor N-PAC | DNA-binding | Positive regulation of histone acetylation, Positive regulation of transcription by RNA polymerase II |
| GNL3 | Guanine nucleotide-binding protein-like 3 | RNA-binding | Cell population proliferation,  Stem cell division |
| GOLGB1 | Golgin subfamily B member 1 | RNA binding, Sequence-specific DNA binding | Golgi organization, Regulation of DNA-templated transcription, Protein localization to pericentriolar material |
| GPTC4 | G patch domain-containing protein 4 | Nucleic acid binding, RNA binding, Protein binding, |  |
| GRSF1 | G-rich sequence factor 1 | RNA-binding | mRNA processing, tRNA processing |
| GRWD1 | Glutamate-rich WD repeat-containing protein 1 | Chromatin binding, DNA replication origin binding, RNA binding, Protein binding | DNA replication, Nucleosome assembly, Nucleosome disassembly, Ribosome biogenesis |
| GTPBP4 | GTP-binding protein 4 | GTP binding, RNA binding | Ribosome biogenesis |
| H2A | Histone H2A type 2-A | DNA-binding |  |
| H2B | Histone H2B type 1-H | DNA-binding | Nucleosome assembly |
| H3 | Histone H3.3C | DNA-binding | Positive regulation of cell growth |
| H4 | Histone H4 | DNA-binding | Nucleosome assembly, Telomere organization |
| HBA | Hemoglobin subunit alpha | Peroxidase activity, Haptoglobin binding, Oxygen carrier activity, Iron ion binding, Protein binding, Oxygen binding, Heme binding, Organic acid binding, Metal ion binding | Carbon dioxide transport, Oxygen transport, Nitric oxide transport, Response to hydrogen peroxide, Hydrogen peroxide catabolic process, Cellular oxidant detoxification, |
| HBB | Hemoglobin subunit beta | Hypotensive agent, Vasoactive | Oxygen transport, Transport |
| HIST2H2AC | Histone H2A type 2-C | DNA-binding |  |
| HIST2H2BE | Histone H2B type 2-E | Antibiotic activity, Antimicrobial activity, DNA-binding | Antibacterial humoral response, Nucleosome assembly |
| HIST2H3C | Histone H3.2 | DNA-binding | Nucleosome assembly |
| HIST4H4 | Histone H4 | DNA-binding | Nucleosome assembly, Telomere organization |
| HLAB | HLA class I histocompatibility antigen  B alpha chain | Chaperone binding, Peptide antigen binding, Signaling receptor binding | Adaptive immunity, Host-virus interaction, Immunity, Innate immunity |
| HMGB1 | High mobility group protein B1 | DNA-binding | Adaptive immunity, Autophagy, Chemotaxis, DNA damage, DNA recombination, DNA repair, Immunity, Inflammatory response, Innate immunity, Host-virus interaction |
| HNRL1 | Heterogeneous nuclear ribonucleoprotein U-like protein 1 | RNA binding, Protein binding | RNA processing, Response to virus |
| HNRL2 | Heterogeneous nuclear ribonucleoprotein U-like protein 2 | RNA binding |  |
| HNRNPK | Heterogeneous nuclear ribonucleoprotein K | Activator, DNA-binding, Repressor, Ribonucleoprotein, RNA-binding | Host-virus interaction, mRNA processing, mRNA splicing, Transcription, Transcription regulation |
| HNRPQ | Heterogeneous nuclear ribonucleoprotein Q | Nucleic acid binding, RNA binding, Protein binding, mRNA 5'-UTR binding | RNA processing, Regulation of translation, RNA splicing, Cellular response to type II interferon |
| HNRPR | Heterogeneous nuclear ribonucleoprotein R | Nucleic acid binding, RNA binding, mRNA binding, Protein binding | mRNA splicing via spliceosome, mRNA processing, RNA splicing |
| HP1B3 | Heterochromatin protein 1-binding protein 3 | DNA binding, Protein binding, Nucleosome binding | Nucleosome assembly, Regulation of DNA-templated transcription, Regulation of cell population proliferation, Heterochromatin organization |
| HRK | Activator of apoptosis harakiri |  | Apoptosis |
| HSPA5 | Endoplasmic reticulum chaperone BiP | Chaperone, Hydrolase activity | Host-virus interaction |
| IF2B2 | Insulin-like growth factor 2 mRNA-binding protein 2 | Nucleic acid binding, RNA binding, mRNA 3'-UTR binding, Protein binding, Translation regulator activity | Regulation of translation, Regulation of gene expression, mRNA transport |
| IF2B3 | Insulin-like growth factor 2 mRNA-binding protein 3 | RNA binding, mRNA 3'-UTR binding, Protein binding, Translation regulator activity | Regulation of cytokine production, Translation, Regulation of translation, Regulation of gene expression, mRNA transport |
| IFI16 | Gamma-interferon-inducible protein 16 | Activator, DNA-binding, Repressor | Apoptosis, Autophagy, Immunity, Inflammatory response, Transcription |
| ILF2 | Interleukin enhancer-binding factor 2 | DNA binding, RNA binding, double-stranded RNA binding, protein binding | Positive regulation of DNA-templated transcription, |
| ILF3 | Interleukin enhancer-binding factor 3 | Virus receptor activity, DNA binding, RNA binding, double-stranded RNA binding, single-stranded RNA binding, protein binding, mRNA 3'-UTR AU-rich region binding | Protein phosphorylation, Regulation of translation, Regulation of viral genome replication, Regulation of DNA-templated transcription, Symbiont entry into host cell, Defense response to virus |
| IMPDH2 | Inosine-5'-monophosphate dehydrogenase 2 | DNA-binding, Oxidoreductase, RNA-binding | GMP biosynthesis, Purine biosynthesis |
| IRAK1 | Interleukin-1 receptor-associated kinase 1 | Kinase activity, Serine/threonine-protein kinase activity, Transferase activity | Host-virus interaction, Innate immunity, Immunity |
| IRAK2 | Interleukin-1 receptor-associated kinase-like 2 | ATP binding, Protein kinase activity, Protein serine/threonine kinase activity | Cytokine-mediated signaling pathway, inflammatory response, Protein phosphorylation |
| IRAK3 | Interleukin-1 receptor-associated kinase 3 | ATP binding, Protein homodimerization activity, Protein heterodimerization activity | Intracellular signal transduction, Protein autophosphorylation, Response to exogenous dsRNA, Response to virus |
| IRAK4 | Interleukin-1 receptor-associated kinase 4 | Kinase activity, Serine/threonine-protein kinase activity, Transferase activity | Innate immunity, Immunity |
| KAT2B | Histone acetyltransferase KAT2B | Activator, Acyltransferase activity, Transferase activity | Biological rhythms, Host-virus interaction, Cell cycle, Transcription, Transcription regulation |
| KNOP1 | Lysine-rich nucleolar protein 1 | RNA binding, Protein binding, |  |
| KPNA1 | Importin subunit alpha-5 | Nuclear import signal receptor activity, Nuclear localization sequence binding | Host-virus interaction, Protein transport |
| KRI1 | Protein KRI1 homolog | RNA-binding |  |
| KRR1 | KRR1 small subunit processome component homolog | Ribonucleoprotein, RNA-binding | Ribosome biogenesis, rRNA processing |
| LAR1B | La-related protein 1B | RNA cap binding, RNA binding, Protein binding | Positive regulation of translation, mRNA stabilization |
| LARP1 | La-related protein 1 | RNA cap binding, RNA 7-methylguanosine cap binding, RNA binding, mRNA 3'-UTR binding, Protein binding, Ribosomal small subunit binding, Cadherin binding | Translation, Translational initiation, Regulation of translation, Post-transcriptional regulation of gene expression, Regulation of macroautophagy, Regulation of viral genome replication |
| LARP4 | La-related protein 4 | RNA binding, Protein binding | Translation, Cytoskeleton organization, Post-transcriptional Regulation of gene expression, Regulation of cell morphogenesis |
| LARP7 | La-related protein 7 | RNA binding, mRNA binding, Protein binding | Regulation of transcription by RNA polymerase II, RNA processing, RNA splicing, Cell differentiation, Regulation of viral transcription |
| LCK | Tyrosine-protein kinase Lck | Kinase activity, Transferase activity, Tyrosine-protein kinase activity | Host-virus interaction |
| LRRFIP1 | Leucine-rich repeat flightless-interacting protein 1 | DNA-binding, Repressor | Transcription, Transcription regulation |
| LRRK2 | Leucine-rich repeat serine/threonine-protein kinase 2 | GTPase activation, Hydrolase activity, Kinase activity, Serine/threonine-protein kinase activity, Transferase activity | Autophagy, Differentiation |
| LYRIC | Protein LYRIC | Transcription coactivator activity, RNA binding, Double-stranded RNA binding, Protein binding, NF-kappaB binding, RNA polymerase II-specific DNA-binding, Transcription factor binding | Regulation of transcription by RNA polymerase II, Regulation of autophagy, Regulation of apoptotic process, Regulation of angiogenesis |
| MBB1A | Myb-binding protein 1A | Activator, Repressor | Biological rhythms, Ribosome biogenesis, Transcription, Transcription regulation |
| MBP | Myelin basic protein | Calmodulin binding, Lipid binding, protease binding, Structural constituent of myelin sheath | Aging, Central nervous system development, Immune response, Response to tumor necrosis factor |
| MFAP3L | Microfibrillar-associated protein 3-like |  |  |
| MMTA2 | Multiple myeloma tumor-associated protein 2 | RNA binding, Protein binding, |  |
| MOV10 | Helicase MOV-10 | Helicase activity, Hydrolase activity, RNA-binding | Host-virus interaction, RNA-mediated gene silencing, Transcription, Transcription regulation |
| MPRIP | Myosin phosphatase Rho-interacting protein | Actin-binding |  |
| MRIP | Myosin phosphatase Rho-interacting protein | Actin-binding |  |
| MRPL38 | 39S ribosomal protein L38, mitochondrial | Ribonucleoprotein, Ribosomal protein |  |
| MRPL44 | 39S ribosomal protein L44, mitochondrial | Endonuclease activity, Hydrolase activity, Nuclease activity, Ribonucleoprotein, Ribosomal protein, RNA-binding |  |
| MRPL47 | 39S ribosomal protein L47, mitochondrial | Ribonucleoprotein, Ribosomal protein |  |
| MRPS17 | 28S ribosomal protein S17, mitochondrial | Ribonucleoprotein, Ribosomal protein, RNA-binding, rRNA-binding |  |
| MRPS35 | 28S ribosomal protein S35, mitochondrial | Ribonucleoprotein, Ribosomal protein |  |
| MRPS7 | 28S ribosomal protein S7, mitochondrial | Ribonucleoprotein, Ribosomal protein |  |
| MSI1H | RNA-binding protein Musashi homolog 1 | Nucleic acid binding, RNA binding, Protein binding, | Regulation of translation, Nervous system development, Response to hormone, Epithelial cell differentiation |
| MSI2H | RNA-binding protein Musashi homolog 2 | Nucleic acid binding, RNA binding, Protein binding, | Regulation of translation, Nervous system development, Stem cell development |
| MTF1 | Metal regulatory transcription factor 1 | Activator, DNA-binding | Transcription, Transcription regulation |
| MX1 | Interferon-induced GTP-binding protein Mx1 | GTP-binding, Protein binding, Microtubule binding | Antiviral defense, Immunity, Innate immunity |
| NAP1L1 | Nucleosome assembly protein 1-like 1 | Chromatin binding, Histone binding, RNA binding | Neurogenesis |
| NCBP1 | Nuclear cap-binding protein subunit 1 | RNA cap binding, RNA 7-methylguanosine cap binding, mRNA binding, Protein binding | mRNA capping, mRNA processing, mRNA splicing, mRNA transport, Nonsense-mediated mRNA decay, RNA-mediated gene silencing, Translation regulation, Transport |
| NCBP2 | Nuclear cap-binding protein subunit 2 | RNA binding, RNA cap binding, DNA binding, Protein binding | mRNA processing, mRNA splicing, mRNA transport, Nonsense-mediated mRNA decay, RNA-mediated gene silencing, Translation regulation, Transport |
| NCBP3 | Nuclear cap-binding protein subunit 3 | RNA-binding |  |
| NCL | Nucleolin | DNA-binding, RNA-binding | Angiogenesis, Cellular response to epidermal growth factor stimulus |
| NKRF | NF-kappa-B-repressing factor | DNA-binding, Repressor | Transcription, Transcription regulation |
| NLRP2 | NACHT, LRR and PYD domains-containing protein 2 | ATP binding, Pyrin domain binding | Apoptosis, Immunity, Inflammatory response, Innate immunity |
| NOC3L | **Nucleolar complex protein 3 homolog** | Chromatin binding, RNA binding | DNA replication initiation, Fat cell differentiation |
| NOG1 | GTP-binding protein 4 | RNA binding, GTPase activity, Protein binding, Preribosome binding | Regulation of cyclin-dependent protein serine/threonine kinase activity, Regulation of DNA replication, Regulation of cell population proliferation, Ribosome biogenesis |
| NOM1 | Nucleolar MIF4G domain-containing protein 1 | RNA binding, Protein binding | Ribosomal small subunit biogenesis, Hair follicle maturation |
| NSUN2 | RNA cytosine C(5)-methyltransferase NSUN2 | Methyltransferase, RNA-binding, Transferase, tRNA-binding | Cell cycle, Cell division, Differentiation, Mitosis, Spermatogenesis, tRNA processing |
| OS9 | Protein OS-9 | Carbohydrate binding, Protease binding | Protein targeting, Response to endoplasmic reticulum stress, Ubiquitin-dependent protein catabolic process |
| PATL1 | Protein PAT1 homolog 1 | RNA binding,protein binding,poly(U) RNA binding,poly(G) binding | Deadenylation-dependent decapping of nuclear-transcribed mRNA, P-body assembly,P-body assembly |
| PCP4L1 | Purkinje cell protein 4-like protein 1 |  |  |
| PDCD5 | Programmed cell death protein 5 | Heparin binding, Acetyltransferase activator activity, Beta-tubulin binding, DNA binding | Apoptosis |
| PEX19 | Peroxisomal biogenesis factor 19 | Protein binding, Peroxisome membrane targeting sequence binding, ATPase binding, Protein carrier chaperone | Peroxisomal biogenesis, Regulation of lipid binding |
| PHF6 | PHD finger protein 6 | DNA, RNA, protein, tubulin, enzyme & histone binding, Histone deacetylase binding, Ribonucleoprotein complex binding, Metal ion binding, Phosphoprotein binding, Scaffold protein binding | Regulation of transcription by RNA polymerase II |
| PKDCC | Extracellular tyrosine-protein kinase PKDCC | Developmental protein, Kinase activity, Transferase activity, Tyrosine-protein kinase activity | Differentiation, Osteogenesis, Protein transport, Transport |
| PLG | Plasminogen | Hydrolase activity, Protease activity, Serine protease activity | Blood coagulation, Fibrinolysis, Hemostasis, Tissue remodeling |
| PLIN3 | Perilipin-3 | Cadherin binding | Transport |
| PMAIP1 | Phorbol-12-myristate-13-acetate-induced protein 1 | Protein binding | Apoptotic process, Elease of cytochrome c from mitochondria, DNA damage response, Regulation of glucose metabolic process, T cell homeostasis |
| POLRMT | DNA-directed RNA polymerase, mitochondrial | Nucleotidyltransferase activity, Transferase activity | Transcription |
| PP1B | Serine/threonine-protein phosphatase PP1-beta catalytic subunit | Protein binding, Hydrolase activity, Phosphatase activity | MAPK cascade, Protein dephosphorylation, Regulation of cell adhesion |
| PPM1G | Protein phosphatase 1G | Hydrolase activity, Protein phosphatase activity, Protein binding | Protein dephosphorylation, Regulation of cell cycle |
| PR40A | Pre-mRNA-processing factor 40 homolog A | RNA binding, Protein binding | mRNA splicing, via spliceosome,  mRNA processing, RNA splicing  Cell migration, Regulation of cytokinesis |
| PRKCE | Protein kinase C epsilon type | Kinase activity, Serine/threonine-protein kinase activity, Transferase activity | Cell adhesion, Cell cycle, Cell division, Immunity |
| PRR3 | Proline-rich protein 3 | RNA binding, Protein binding, Metal ion binding |  |
| PSMC1 | 26S proteasome regulatory subunit 4 | ATP binding, RNA binding, Proteasome-activating activity | Negative regulation of neuron death, proteasome-mediated ubiquitin-dependent protein catabolic process |
| PTCD3 | Small ribosomal subunit protein mS39 | RNA binding, Protein binding, rRNA binding, Ribosomal small subunit binding | Regulation of translation, Mitochondrial translation |
| PUF60 | Poly(U)-binding-splicing factor PUF60 | DNA-binding, Repressor, Ribonucleoprotein, RNA-binding | Apoptosis, mRNA processing, mRNA splicing, Transcription, Transcription regulation |
| PURA | Transcriptional activator protein Pur-alpha | mRNA regulatory element binding, Translation repressor activity, DNA-binding transcription factor activity, Protein binding, SMAD binding, Transcription regulator inhibitor activity | Regulation of transcription,  DNA replication initiation |
| PURB | Transcriptional activator protein Pur-beta | DNA-binding transcription factor activity, RNA binding, mRNA binding, Protein binding, SMAD binding | Regulation of transcription |
| PWP1 | Periodic tryptophan protein 1 homolog | Histone chaperone activity, H4K20me3 modified histone binding, | DNA-templated transcription, rRNA processing, Regulation of peptidyl-serine phosphorylation of STAT protein, Ribosome biogenesis |
| PYM1 | Partner of Y14 and mago |  |  |
| PYM1 | Partner of Y14 and mago | RNA binding, Protein binding, Ribosome binding | Regulation of translation, Exon-exon junction complex disassembly |
| RBM4 | RNA-binding protein 4 | RNA binding, mRNA binding, mRNA 3'-UTR binding, Protein binding, Cyclin binding, Metal ion binding | Cap-independent translational initiation, RNA processing, RNA splicing, Regulation of gene expression |
| RBMS1 | RNA-binding motif, single-stranded-interacting protein 1 | DNA binding, RNA binding, mRNA 3'-UTR binding, Protein binding | DNA replication, RNA processing |
| RFC1 | Replication factor C subunit 1 | DNA binding, Protein binding, ATP binding, ATP hydrolysis activity | DNA replication, DNA repair, Telomere maintenance, DNA clamp unloading |
| RFC2 | Replication factor C subunit 2 | DNA clamp loader activity, DNA helicase activity, ATP hydrolysis activity, Enzyme binding | DNA replication, DNA repair, Regulation of DNA-directed DNA polymerase activity |
| RFC4 | Replication factor C subunit 4 | DNA clamp loader activity, DNA helicase activity, ATP hydrolysis activity, Enzyme binding | DNA replication, DNA repair, Regulation of DNA-directed DNA polymerase activity |
| RILPL2 | RILP-like protein 2 | Protein binding, Protein dimerization activity | Protein transport, transport |
| RL1D1 | Ribosomal L1 domain-containing protein 1 | Regulation of protein localization, RNA binding, mRNA 3'-UTR binding, protein binding | Regulation of apoptotic process, Regulation of cellular senescence |
| RL5 | Large ribosomal subunit protein uL18 | RNA binding, mRNA 3'-UTR binding, Structural constituent of ribosome, Protein binding | Cytoplasmic translation, rRNA processing, Regulation of gene expression, Ribosomal large subunit biogenesis, Regulation of translation, Regulation of ubiquitin protein ligase activity |
| RL6 | Large ribosomal subunit protein eL6 | DNA binding, RNA binding, Structural constituent of ribosome, Protein binding, Cadherin binding | Cytoplasmic translation, regulation of DNA-templated transcription, translation |
| RM01 | Large ribosomal subunit protein uL1m | RNA binding, Structural constituent of ribosome, Protein binding | Translation, Mitochondrial translation |
| RM02 | Large ribosomal subunit protein uL2m | RNA binding, Structural constituent of ribosome, Protein binding | Translation, Mitochondrial translation |
| RM03 | Large ribosomal subunit protein uL3m | RNA binding, Structural constituent of ribosome, Protein binding | Translation, Mitochondrial translation |
| RM04 | Large ribosomal subunit protein uL4m | RNA binding, Structural constituent of ribosome, Protein binding | Translation, Mitochondrial translation |
| RM09 | Large ribosomal subunit protein bL9m | RNA binding, Structural constituent of ribosome, Protein binding | Translation, Mitochondrial translation |
| RM15 | Large ribosomal subunit protein uL15m | RNA binding, Structural constituent of ribosome, Protein binding | Translation, Mitochondrial translation, Cellular response to leukemia inhibitory factor |
| RM18 | Large ribosomal subunit protein uL18m | RNA binding, Structural constituent of ribosome, Protein binding | Translation, Mitochondrial translation, rRNA import into mitochondrion |
| RM19 | Large ribosomal subunit protein bL19m | Structural constituent of ribosome, Protein binding, | Translation, Mitochondrial translation |
| RM20 | Large ribosomal subunit protein bL20m | RNA binding, Structural constituent of ribosome, Protein binding, rRNA binding | Translation, Mitochondrial translation |
| RM21 | Large ribosomal subunit protein bL21m | RNA binding, Structural constituent of ribosome | Translation, Mitochondrial translation |
| RM22 | Large ribosomal subunit protein uL22m | RNA binding, Structural constituent of ribosome | Translation, Mitochondrial translation |
| RM23 | Large ribosomal subunit protein uL23m | RNA binding, Structural constituent of ribosome | Translation, Mitochondrial translation |
| RM24 | Large ribosomal subunit protein uL24m | RNA binding, Structural constituent of ribosome | Translation, Mitochondrial translation |
| RM28 | Large ribosomal subunit protein bL28m | RNA binding, Structural constituent of ribosome | Translation, Mitochondrial translation |
| RM32 | Large ribosomal subunit protein bL32m | RNA binding, Structural constituent of ribosome | Translation, Mitochondrial translation |
| RM37 | Large ribosomal subunit protein mL37 | RNA binding, Structural constituent of ribosome | Translation, Mitochondrial translation |
| RM38 | Large ribosomal subunit protein mL38 | Protein binding, | Mitochondrial translation |
| RM39 | Large ribosomal subunit protein mL39 | Nucleotide binding, RNA binding, | Mitochondrial translation |
| RM41 | Large ribosomal subunit protein mL41 | RNA binding, Structural constituent of ribosome, Protein binding | Translation, Apoptotic process, Cell cycle, Mitochondrial translation |
| RM43 | Large ribosomal subunit protein mL43 | RNA binding, Structural constituent of ribosome, Protein binding | Translation, Mitochondrial translation |
| RM44 | Large ribosomal subunit protein mL44 | Ribonuclease III activity, RNA binding, double-stranded RNA binding, Nuclease activity, Endonuclease activity, Protein binding, Hydrolase activity | RNA processing, Gene expression, Mitochondrial translation, Mitochondrial translational elongation |
| RM45 | Large ribosomal subunit protein mL45 | RNA binding, Protein binding | Mitochondrial translation |
| RM46 | Large ribosomal subunit protein mL46 | Structural constituent of ribosome | Mitochondrial translation |
| RM47 | Large ribosomal subunit protein uL29m | Structural constituent of ribosome | Translation, Mitochondrial translation |
| RM48 | Large ribosomal subunit protein mL48 | Protein binding, | Mitochondrial translation |
| RM49 | Large ribosomal subunit protein mL49 | Structural constituent of ribosome, Protein binding, | Translation, Mitochondrial translation |
| RM50 | Large ribosomal subunit protein mL50 | Ribonucleoprotein, Ribosomal protein | Mitochondrial translation |
| RM54 | Large ribosomal subunit protein mL54 | RNA binding, structural constituent of ribosome, Protein binding, | Mitochondrial translation, |
| RM55 | Large ribosomal subunit protein mL55 | Structural constituent of ribosome | Translation, Mitochondrial translation |
| RPF2 | Ribosome production factor 2 homolog | RNA binding, protein binding, rRNA binding |  |
| RPL12 | 60S ribosomal protein L12 | Ribonucleoprotein, Ribosomal protein, RNA-binding | Cytoplasmic translation, |
| RPL5 | 60S ribosomal protein L5 | Ribonucleoprotein, Ribosomal protein, RNA-binding, rRNA-binding | Cytoplasmic translation, Protein stabilization, rRNA processing |
| RPL6 | 60S ribosomal protein L6 | Ribonucleoprotein, Ribosomal protein | Cytoplasmic translation, DNA-templated transcription, Ribosomal large subunit assembly |
| RPOM | DNA-directed RNA polymerase, mitochondrial | 3'-5'-RNA exonuclease activity, DNA binding, RNA binding , DNA primase activity, Protein binding, Transferase activity | DNA replication, Synthesis of RNA primer, DNA-templated transcription, Mitochondrial transcription |
| RPS27 | 40S ribosomal protein S27 | Ribonucleoprotein, Ribosomal protein | Cytoplasmic translation, rRNA processing, ribosomal small subunit assembly |
| RPS7 | 40S ribosomal protein S7 | Ribonucleoprotein, Ribosomal protein | Cytoplasmic translation, rRNA processing |
| RRP1 | Ribosomal RNA processing protein 1 homolog A |  | rRNA processing |
| RRP12 | RRP12-like protein | RNA binding | rRNA processing |
| RRP1B | Ribosomal RNA processing protein 1 homolog B | Activator | Apoptosis, Host-virus interaction, mRNA processing, mRNA splicing, Transcription, Transcription regulation |
| RRP5 | Protein RRP5 homolog | RNA binding, protein binding, NF-kappaB binding, | rRNA processing, RNA processing |
| RS7 | Small ribosomal subunit protein eS7 | mRNA 3'-UTR binding, mRNA 5'-UTR binding, Protein kinase binding, RNA binding, Structural constituent of ribosome, Ubiquitin ligase inhibitor activity | Translation, rRNA processing, Regulation of gene expression, Ribosomal small subunit biogenesis, Regulation of intrinsic apoptotic signaling pathway by p53 class mediator |
| RT02 | Small ribosomal subunit protein uS2m | Ribonucleoprotein, Ribosomal protein | Mitochondrial ribosome assembly, Mitochondrial translation |
| RT05 | Small ribosomal subunit protein uS5m | Ribonucleoprotein, Ribosomal protein | Apoptotic process, Mitochondrial translation |
| RT07 | Small ribosomal subunit protein uS7m | mRNA binding, RNA binding, rRNA binding, Structural constituent of ribosome | Ribosomal small subunit assembly, Mitochondrial translation, Translation |
| RT09 | Small ribosomal subunit protein uS9m | Ribonucleoprotein, Ribosomal protein | Mitochondrial translation |
| RT17 | Small ribosomal subunit protein uS17m | Ribonucleoprotein, Ribosomal protein, RNA-binding, rRNA-binding | Mitochondrial translation, Translation |
| RT21 | Small ribosomal subunit protein bS21m | Ribonucleoprotein, Ribosomal protein | Translation, Mitochondrial translation |
| RT22 | Small ribosomal subunit protein mS22 | Ribonucleoprotein, Ribosomal protein | Mitochondrial translation |
| RT25 | Small ribosomal subunit protein mS25 | Ribonucleoprotein, Ribosomal protein | Mitochondrial translation |
| RT26 | Small ribosomal subunit protein mS26 | Ribonucleoprotein, Ribosomal protein | RNA binding, Mitochondrial translation |
| RT27 | Small ribosomal subunit protein mS27 | Ribonucleoprotein, Ribosomal protein, RNA-binding, rRNA-binding, tRNA-binding | Cell population proliferation, Mitochondrial translation, Positive regulation of mitochondrial translation |
| RT29 | Small ribosomal subunit protein mS29 | Ribonucleoprotein, Ribosomal protein | Apoptotic process, Mitochondrial translation |
| RT30 | Large ribosomal subunit protein mL65 | Ribonucleoprotein, Ribosomal protein | Apoptotic process, Mitochondrial translation |
| RT33 | Small ribosomal subunit protein mS33 | Ribonucleoprotein, Ribosomal protein | Translation, Mitochondrial translation |
| RT34 | Small ribosomal subunit protein mS34 | Ribonucleoprotein, Ribosomal protein | Mitochondrial translation |
| RT35 | Small ribosomal subunit protein mS35 | RNA binding, Ribonucleoprotein, Ribosomal protein | Mitochondrial translation |
| S100A9 | Protein S100-A9 | Antimicrobial activity, Antioxidant activity | Apoptosis, Autophagy, Immunity, Inflammatory response, Chemotaxis, Innate immunity |
| S6A17 | Sodium-dependent neutral amino acid transporter SLC6A17 | Symporter activity | Amino-acid transport, Ion transport, Neurotransmitter transport, Sodium transport, Symport, Transport |
| SF3B4 | Splicing factor 3B subunit 4 | RNA-binding | mRNA processing, mRNA splicing |
| SIPA1 | Signal-induced proliferation-associated protein 1 | GTPase activation | Adaptive immune response, Signal transduction |
| SLC24A2 | Sodium/potassium/calcium exchanger 2 | Calcium channel activity, Calcium, potassium/sodium antiporter activity, Symporter activity | Antiport, Calcium transport, Ion transport, Potassium transport, Sodium transport, Symport |
| SMCA5 | SWI/SNF-related matrix-associated actin-dependent regulator of chromatin subfamily A member 5 | Chromatin regulator, Helicase activity, Hydrolase activity | Host-virus interaction |
| SR140 | U2 snRNP-associated SURP motif-containing protein | RNA-binding | RNA processing |
| SRBD1 | S1 RNA-binding domain-containing protein 1 | RNA-binding |  |
| SRP14 | Signal recognition particle 14 kDa protein | Ribonucleoprotein, RNA-binding |  |
| SRPK1 | SRSF protein kinase 1 | Kinase activity, Serine/threonine-protein kinase activity, Transferase activity | Chromosome partition, Differentiation, mRNA processing, mRNA splicing |
| SRS12 | Serine/arginine-rich splicing factor 12 | RNA-binding | mRNA processing, mRNA splicing |
| STAU1 | Double-stranded RNA-binding protein Staufen homolog 1 | RNA-binding | Host-virus interaction |
| STRBP | Spermatid perinuclear RNA-binding protein | Developmental protein, DNA-binding, RNA-binding | Differentiation, Spermatogenesis |
| SYFM | Phenylalanine--tRNA ligase, mitochondrial | Aminoacyl-tRNA synthetase, Ligase | Protein biosynthesis |
| TAF8 | Transcription initiation factor TFIID subunit 8 | Developmental protein | Differentiation, Transcription, Transcription regulation |
| TMF1 | TATA element modulatory factor | DNA-binding, Repressor | Transcription, Transcription regulation |
| TNF | Tumor necrosis factor ligand superfamily member 18 | Cytokine | Adaptive immunity, Immunity |
| TNFRSF9 | Tumor necrosis factor receptor superfamily member 9 | Signaling receptor binding | Apoptotic process, Regulation of cell proliferation, Regulation of T cell proliferation, Cell-cell signaling |
| TNFSF10 | Tumor necrosis factor ligand superfamily member 10 | Cytokine activity | Apoptosis, Immune response |
| TOP1 | DNA topoisomerase 1 | DNA-binding, Isomerase activity, Topoisomeras activity e | Biological rhythms, Host-virus interaction |
| TP73 | Tumor protein p73 | Activator, DNA-binding | Apoptosis, Cell cycle, Host-virus interaction, Transcription, Transcription regulation |
| TRI26 | Tripartite motif-containing protein 26 | Transferase activity | Immunity, Innate immunity |
| TRIM26 | Tripartite motif-containing protein 26 | Transferase activity | Innate immunity |
| TSR1 | Pre-rRNA-processing protein TSR1 homolog | RNA binding, GTPase activity | Ribosome biogenesis |
| TUBA1A | Tubulin alpha-1A chain | Hydrolase activity | Cell division, Cerebral cortex development, Gene expression, Intracellular protein transport |
| UBE2I | SUMO-conjugating enzyme UBC9 | Transferase activity | Cell cycle, Cell division, Host-virus interaction, Chromosome partition, Mitosis, Ubl conjugation pathway |
| WIBG | Partner of Y14 and mago | RNA-binding | Nonsense-mediated mRNA decay, Translation regulation |
| WWP1 | NEDD4-like E3 ubiquitin-protein ligase WWP1 | Transferase activity | Host-virus interaction, Ubl conjugation pathway |
| XRCC5 | X-ray repair cross-complementing protein 5 | Activator, DNA-binding, Helicase activity, Hydrolase activity | Host-virus interaction, DNA damage, DNA recombination, DNA repair, Immunity, Innate immunity, Ribosome biogenesis, Transcription, Transcription regulation |
| XRCC6 | X-ray repair cross-complementing protein 6 | Activator, DNA-binding, Helicase,  Hydrolase, Lyase, Multifunctional enzyme | Host-virus interaction, DNA damage,  DNA recombination, DNA repair, Immunity, Innate immunity, Transcription, Transcription regulation |
| XRN2 | 5'-3' exoribonuclease 2 | DNA-binding, Exonuclease, Hydrolase,, Nuclease | mRNA processing, Transcription, Transcription regulation, Transcription termination |
| YTDC2 | 3'-5' RNA helicase YTHDC2 | Helicase, Hydrolase, RNA-binding | Differentiation, Meiosis, Oogenesis, Spermatogenesis |
| ZAP70 | Tyrosine-protein kinase ZAP-70 | Kinase, Transferase, Tyrosine-protein kinase | Adaptive immunity, Immunity |
| ZC3H8 | Zinc finger CCCH domain-containing protein 8 | Repressor, RNA-binding | Apoptosis, Transcription, Transcription regulation |
| ZCCHV | Zinc finger CCCH-type antiviral protein 1 | RNA-binding, Cadherin binding | Immune system process, Antiviral defense, Immunity, Innate immunity |
| ZCHC3 | Zinc finger CCHC domain-containing protein 3 | DNA-binding, RNA-binding | Antiviral defense, Immunity, Innate immunity |
| ZFR | Zinc finger RNA-binding protein | Developmental protein, DNA-binding, RNA-binding |  |
| ZN394 | Zinc finger protein 394 | DNA-binding | Transcription, Transcription regulation |
| ZNF394 | Zinc finger protein 394 | DNA-binding | Transcription, Transcription regulation |
| ZNF410 | Zinc finger protein 410 | DNA-binding transcription factor activity, Activator, DNA-binding | Transcription, Transcription regulation |

**Table S2. Structural information and docking scores for potential DENV-2 capsid protein inhibitors derived from *Azadirachta indica*.**

| **Compound Number** | **Structure** | **Docking score (kcal/mol)** | **Compound Number** | **Structure** | **Docking score (kcal/mol)** |
| --- | --- | --- | --- | --- | --- |
| AI-181 |  | -10.4 | AI-108 |  | -10.3 |
| AI-158 |  | -10.2 | AI-199 |  | -10.2 |
| AI-84 |  | -10.2 | AI-202 |  | -10.1 |
| AI-87 |  | -10.1 | AI-130 |  | -10 |
| AI-153 |  | -10 | AI-163 |  | -10 |
| AI-79 |  | -10 | AI-149 | 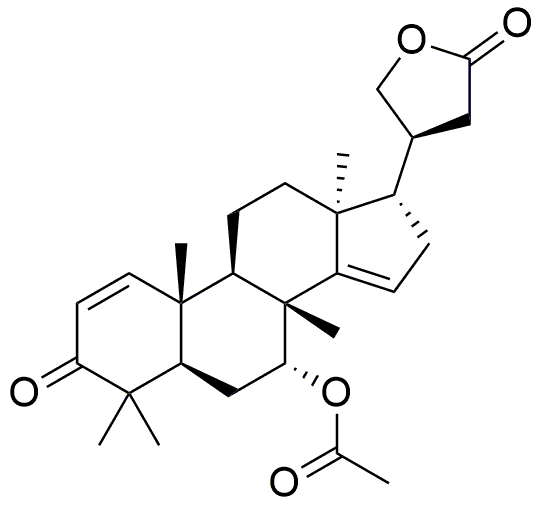 | -9.9 |
| AI-162 | 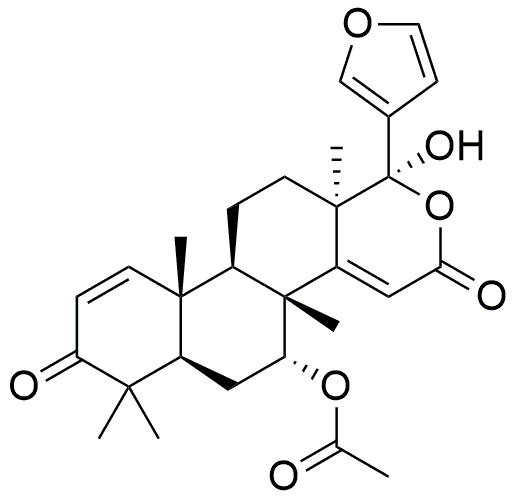 | -9.9 | AI-179 | 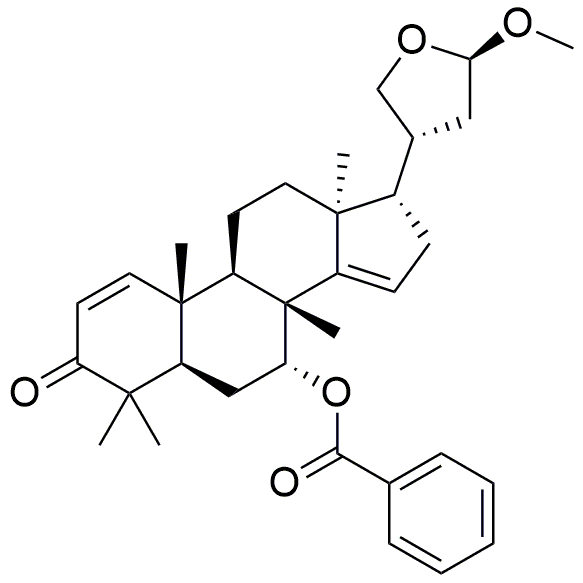 | -9.8 |
| AI-111 | 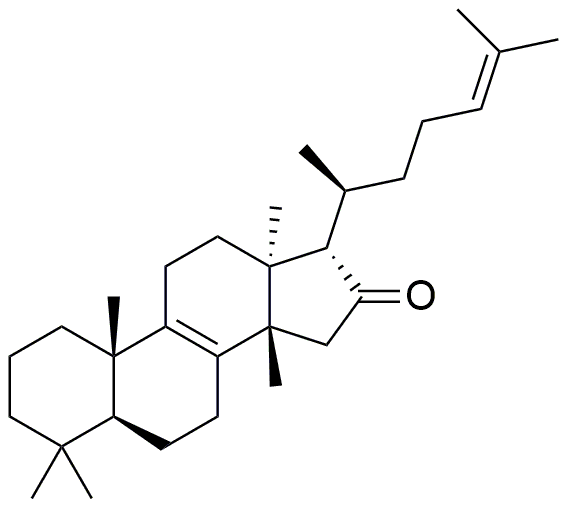 | -9.7 | AI-137 | 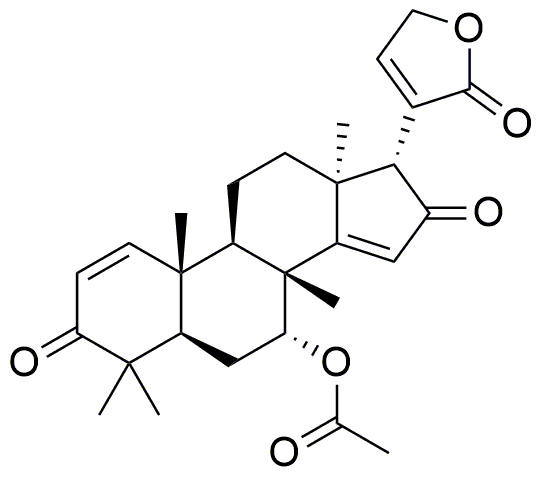 | -9.7 |
| AI-25 | 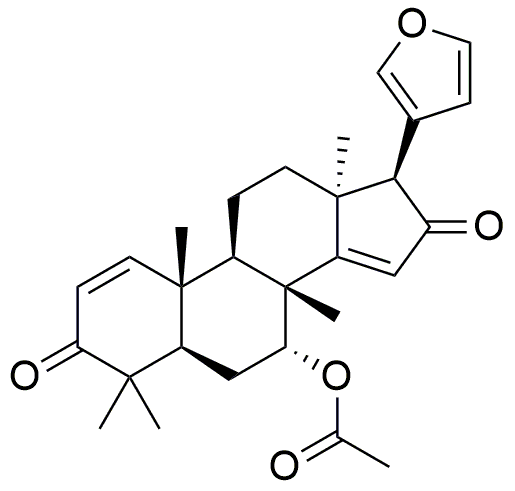 | -9.7 | AI-186 | 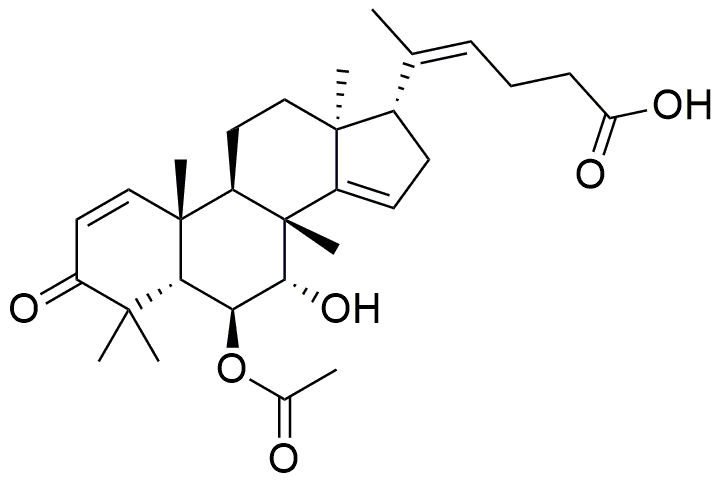 | -9.6 |
| AI-187 | 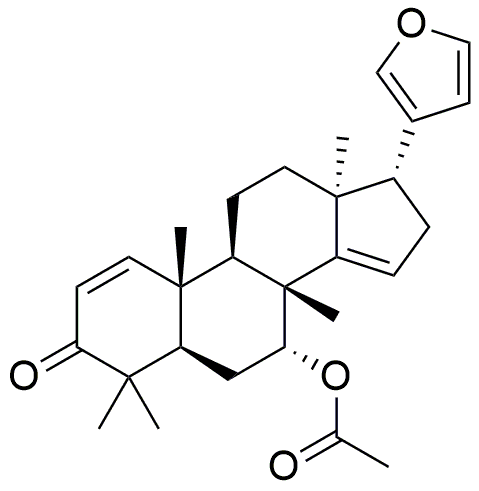 | -9.6 | AI-6 | 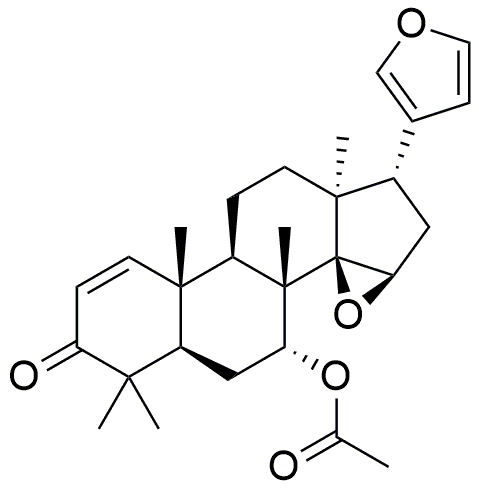 | -9.6 |
| AI-118 |  | -9.5 | AI-159 |  | -9.5 |
| AI-160 |  | -9.5 | AI-193 |  | -9.5 |
| AI-198 | 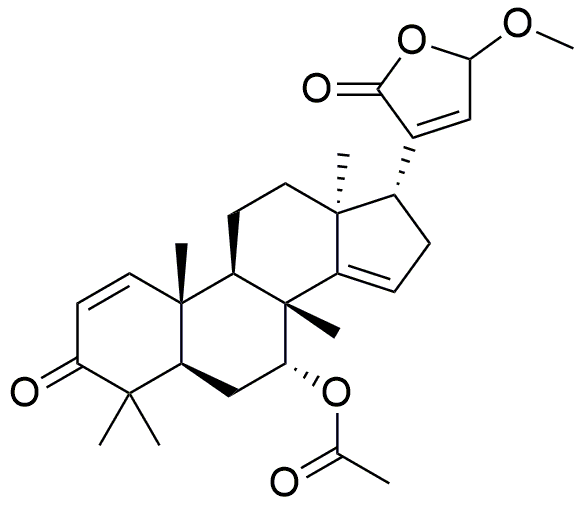 | -9.5 | AI-59 |  | -9.5 |
| AI-103 | 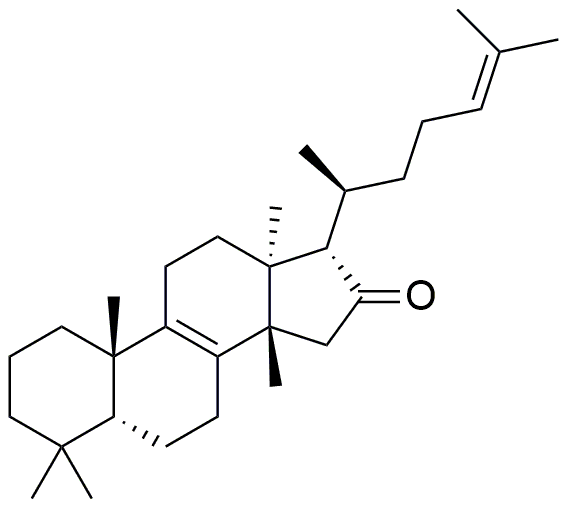 | -9.4 | AI-128 |  | -9.4 |
| AI-138 | 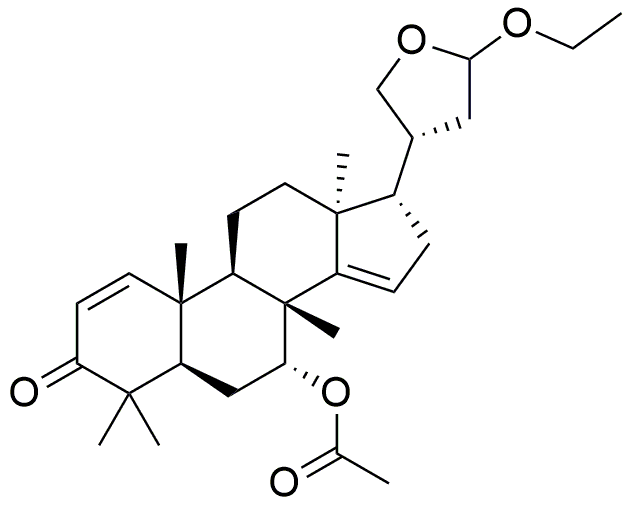 | -9.4 | AI-161 |  | -9.4 |
| AI-24 | 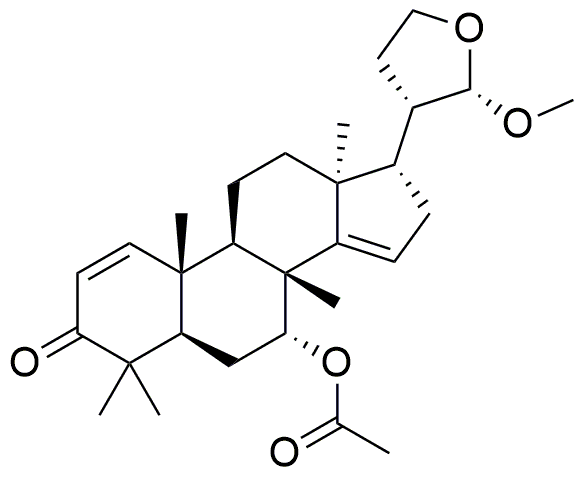 | -9.4 | AI-116 |  | -9.3 |
| AI-117 | 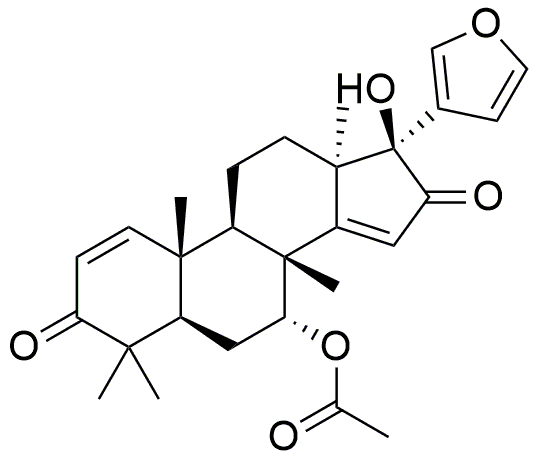 | -9.3 | AI-142 |  | -9.3 |
| AI-157 | 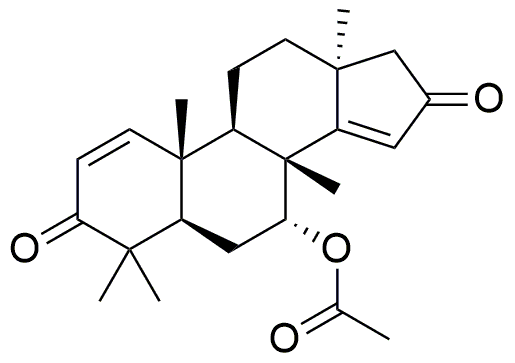 | -9.3 | AI-169 |  | -9.3 |
| AI-185 | 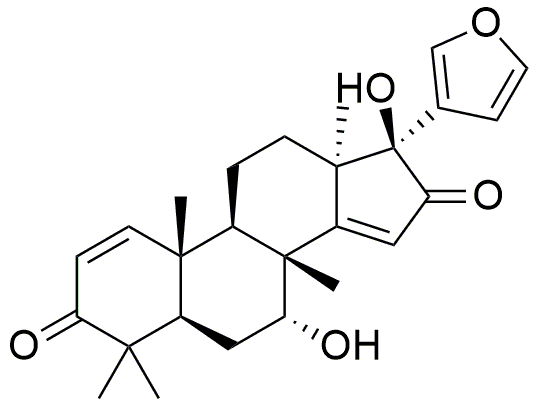 | -9.3 | AI-180 |  | -9.2 |
| AI-203 | 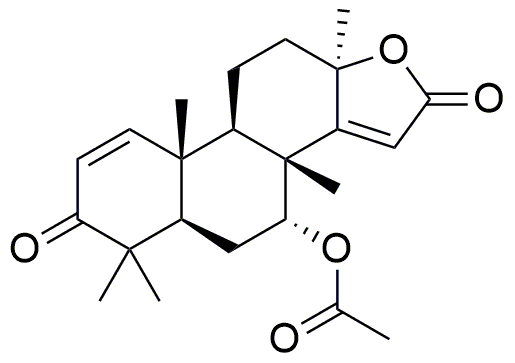 | -9.2 | AI-61 |  | -9.1 |
| AI-65 | 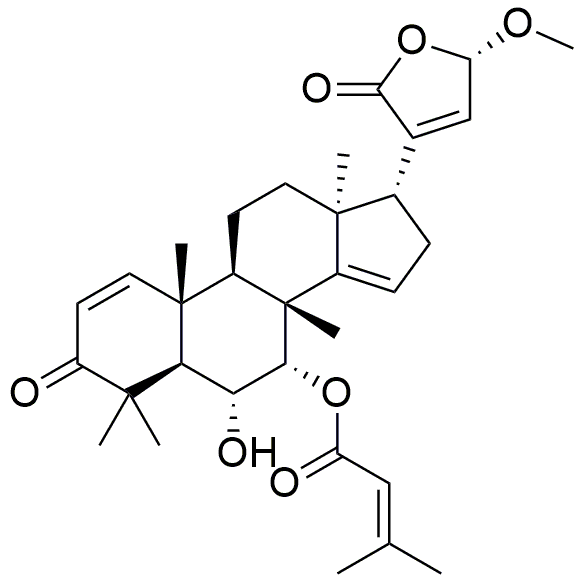 | -9.1 | AI-75 |  | -9.1 |
| AI-91 | 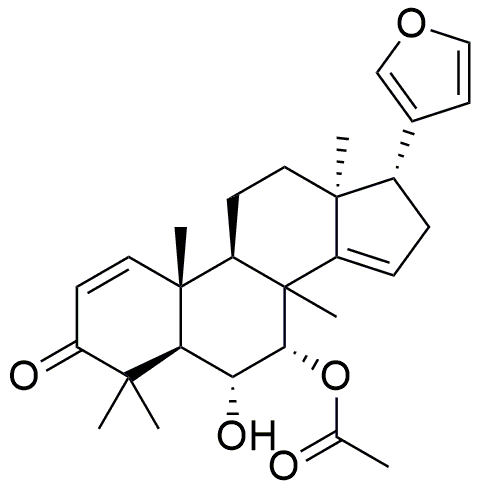 | -9.1 | AI-94 |  | -9.1 |
| AI-115 | 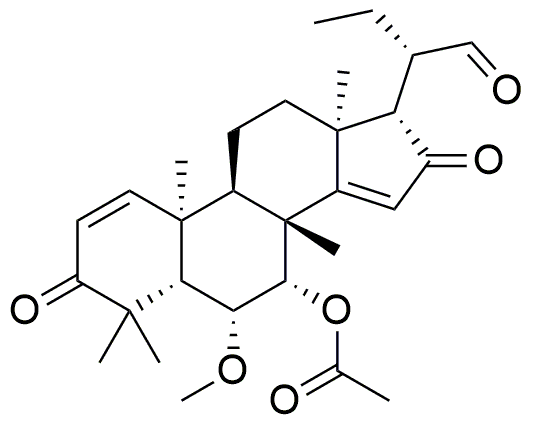 | -9.0 | AI-131 |  | -9.0 |
| AI-140 | 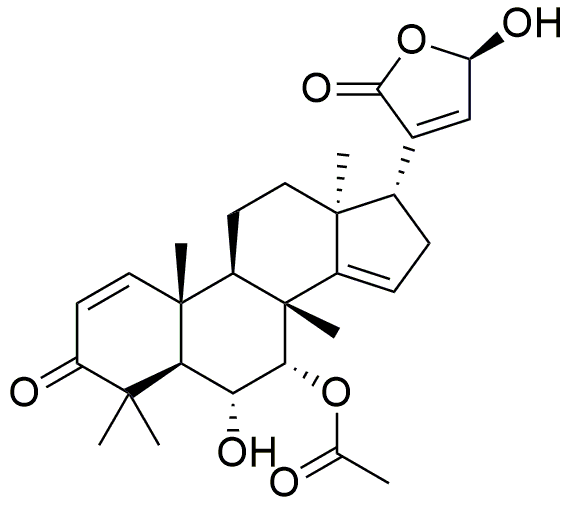 | -9.0 | AI-150 |  | -9.0 |
| AI-164 | 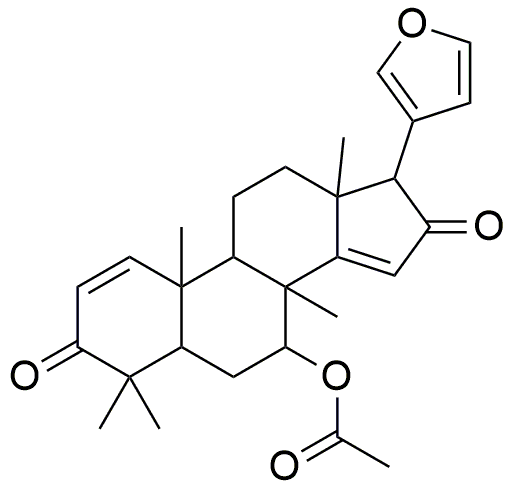 | -9.0 | AI-188 |  | -9.0 |
| AI-27 | 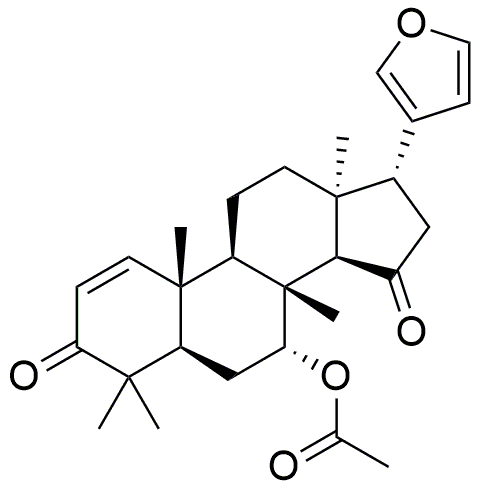 | -9.0 | AI-82 |  | -9.0 |
| AI-110 | 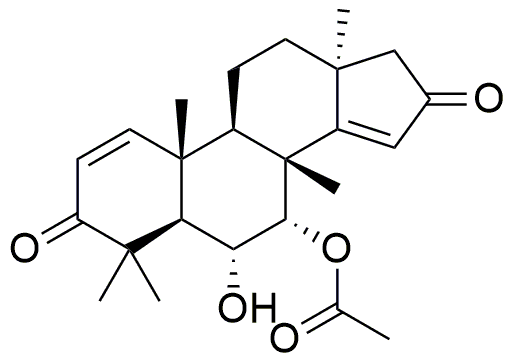 | -8.9 | AI-175 |  | -8.9 |
| AI-189 | 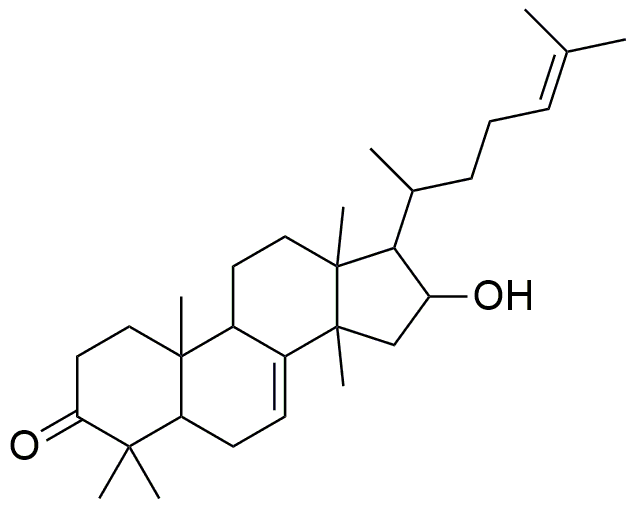 | -8.9 | AI-190 |  | -8.9 |
| AI-36 | 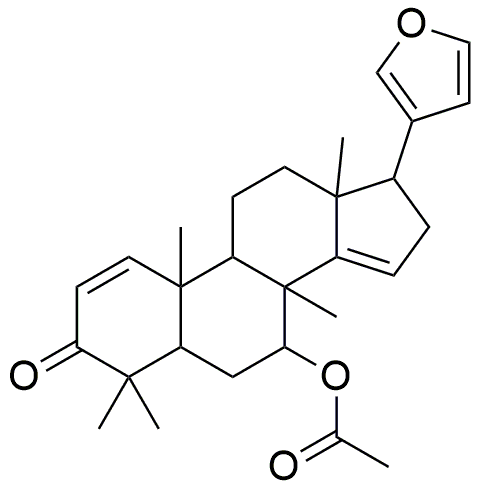 | -8.9 | AI-47 |  | -8.9 |
| AI-71 | 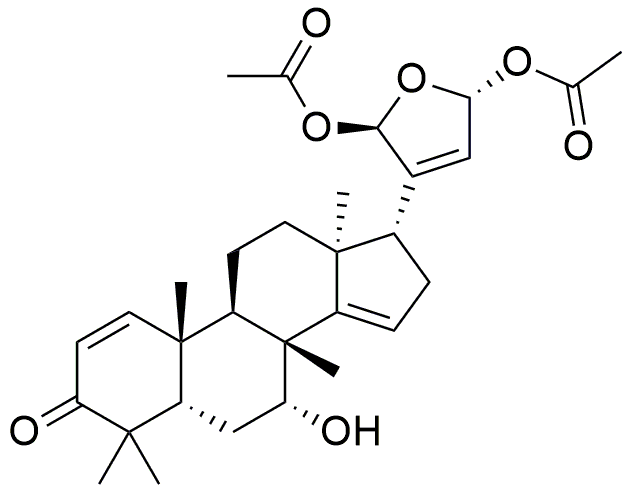 | -8.9 | AI-133 |  | -8.8 |
| AI-166 | 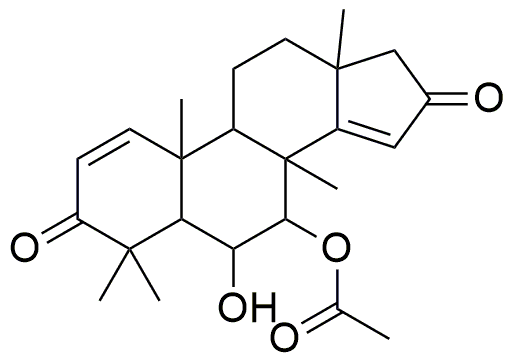 | -8.8 | AI-178 |  | -8.8 |
| AI-30 | 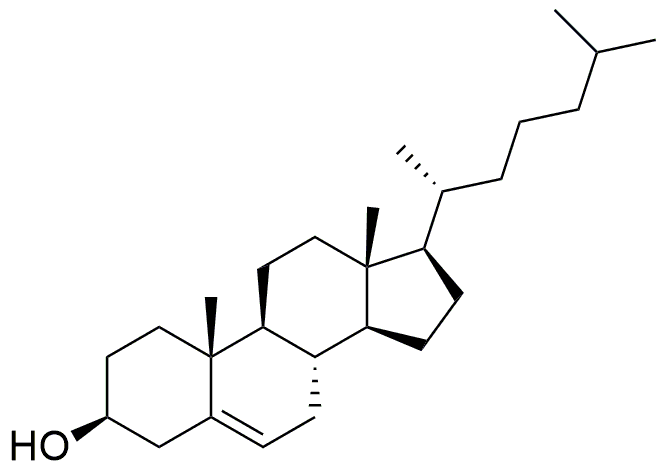 | -8.8 | AI-37 |  | -8.8 |
| AI-78 | 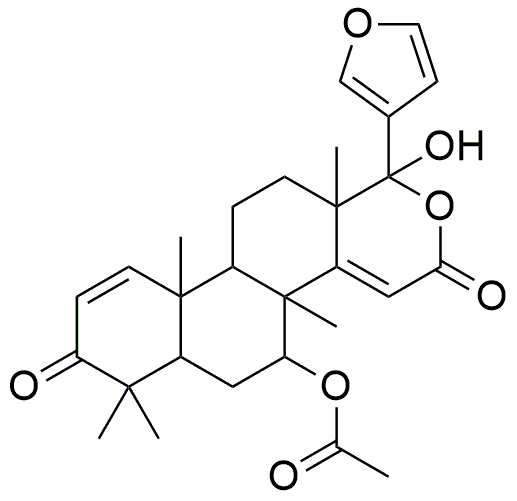 | -8.8 | AI-96 |  | -8.8 |
| AI-121 | 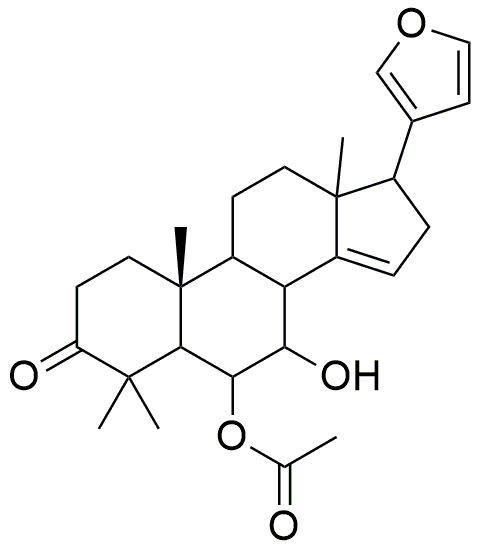 | -8.7 | AI-165 |  | -8.7 |
| AI-40 | 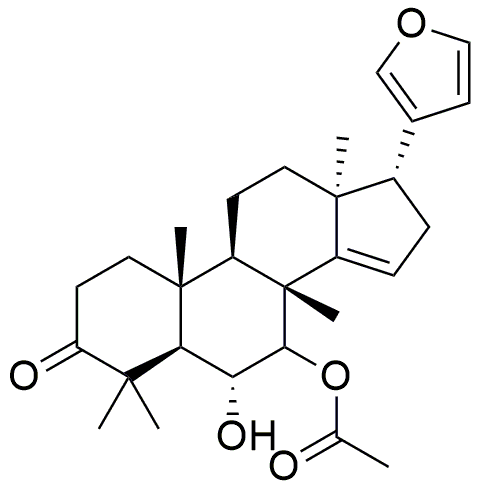 | -8.7 | AI-35 |  | -8.6 |
| AI-45 | 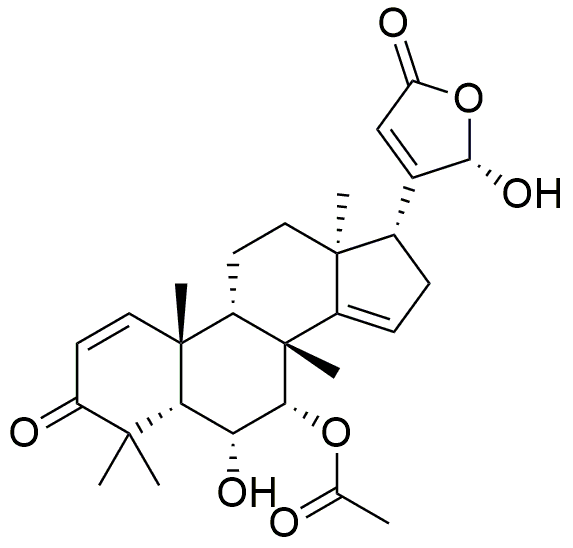 | -8.6 | AI-62 |  | -8.6 |
| AI-64 | 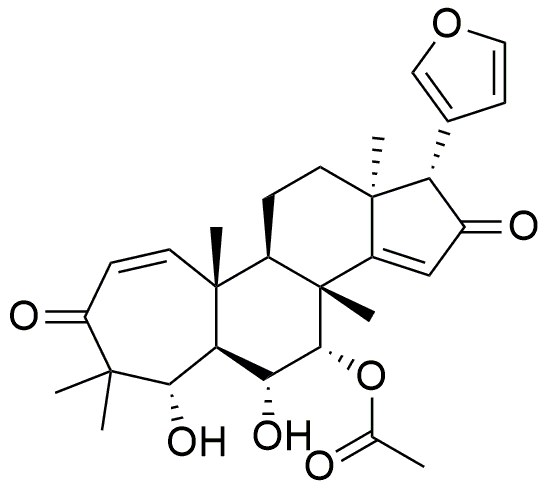 | -8.6 | AI-72 |  | -8.6 |
| AI-88 | 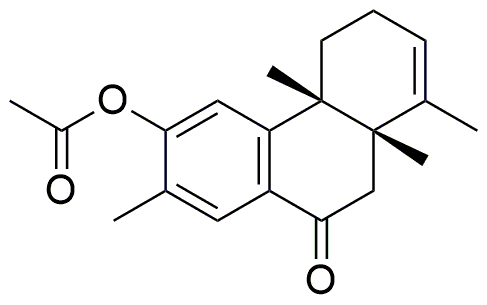 | -8.6 | AI-109 |  | -8.5 |
| AI-136 | 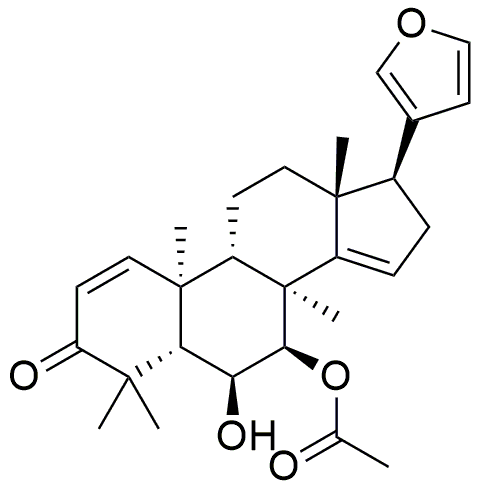 | -8.5 | AI-154 |  | -8.5 |
| AI-176 | 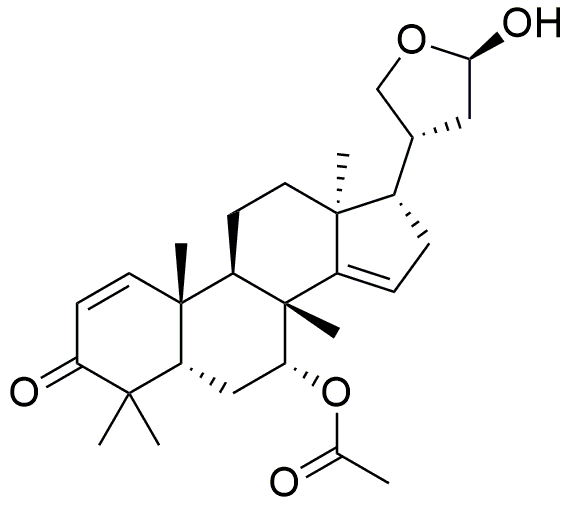 | -8.5 | AI-76 |  | -8.5 |
| AI-93 | 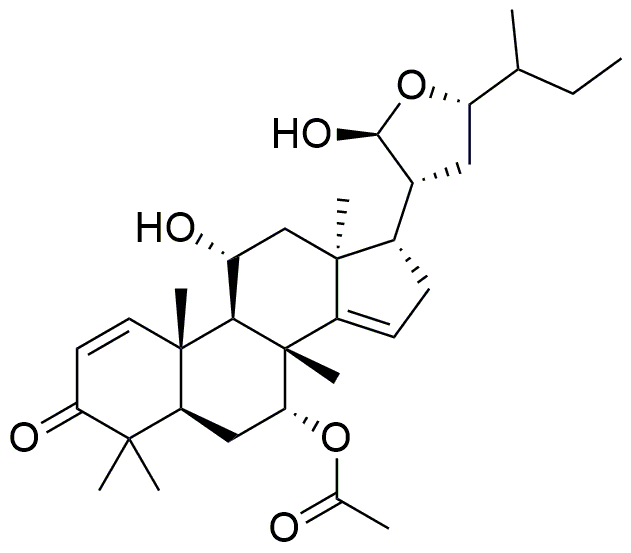 | -8.5 |  |  |  |

**Table S3. Physicochemical characteristics of inhibitors targeting the DENV-2 capsid protein derived from *Azadirachta indica*.**

| **CN** | **MW** | **HA** | **RB** | **HBA** | **HBA** | **TPSA** | **Log** | **SP** | **LV** | **PAINS** | **SA** |
| --- | --- | --- | --- | --- | --- | --- | --- | --- | --- | --- | --- |
| AI-181 | 468.58 | 34 | 3 | 6 | 1 | 89.90 | 3.88 | -6.03 | 0 | 0 | 6.21 |
| AI-108 | 512.64 | 38 | 4 | 5 | 0 | 73.58 | 5.42 | -4.83 | 2 | 0 | 6.01 |
| AI-158 | 452.58 | 33 | 3 | 5 | 1 | 76.74 | 4.16 | -5.70 | 0 | 0 | 6.00 |
| AI-199 | 498.65 | 37 | 4 | 4 | 0 | 56.51 | 6.08 | -4.10 | 1 | 0 | 5.99 |
| AI-84 | 408.53 | 30 | 1 | 4 | 1 | 67.51 | 3.92 | -5.77 | 0 | 0 | 5.71 |
| AI-202 | 542.62 | 39 | 5 | 9 | 2 | 136.43 | 2.58 | -8.04 | 1 | 0 | 6.7 |
| AI-87 | 386.48 | 28 | 2 | 5 | 0 | 69.67 | 3.35 | -6.34 | 0 | 0 | 5.43 |
| AI-130 | 454.6 | 33 | 4 | 5 | 1 | 80.67 | 4.69 | -5.10 | 1 | 0 | 5.92 |
| AI-153 | 468.58 | 34 | 3 | 6 | 1 | 89.90 | 3.81 | -6.35 | 0 | 0 | 6.19 |
| AI-163 | 466.57 | 34 | 3 | 6 | 1 | 93.81 | 4.01 | -5.82 | 0 | 0 | 5.94 |
| AI-79 | 470.64 | 34 | 4 | 5 | 0 | 61.83 | 4.84 | -5.32 | 1 | 0 | 6.42 |
| AI-149 | 454.6 | 33 | 3 | 5 | 0 | 69.67 | 4.50 | -5.51 | 1 | 0 | 5.96 |
| AI-162 | 482.57 | 35 | 3 | 7 | 1 | 103.04 | 3.72 | -6.51 | 0 | 0 | 6.09 |
| AI-179 | 532.71 | 39 | 5 | 5 | 0 | 61.83 | 5.89 | -4.52 | 2 | 0 | 6.41 |
| AI-111 | 424.70 | 31 | 4 | 1 | 0 | 17.07 | 7.53 | -2.63 | 1 | 0 | 6.11 |
| AI-137 | 466.57 | 34 | 3 | 6 | 0 | 86.74 | 3.82 | -6.26 | 0 | 0 | 6.11 |
| AI-25 | 450.57 | 33 | 3 | 5 | 0 | 73.58 | 4.33 | -5.63 | 0 | 0 | 5.89 |
| AI-186 | 498.65 | 36 | 6 | 6 | 2 | 100.9 | 4.48 | -5.73 | 0 | 0 | 6.32 |
| AI-187 | 436.58 | 32 | 3 | 4 | 0 | 56.51 | 5.02 | -4.9 | 1 | 0 | 5.87 |
| AI-6 | 452.58 | 33 | 3 | 5 | 0 | 69.04 | 4.51 | -5.57 | 0 | 0 | 6.32 |
| AI-118 | 468.58 | 34 | 3 | 6 | 1 | 89.27 | 3.67 | -6.37 | 0 | 0 | 6.52 |
| AI-159 | 466.57 | 34 | 3 | 6 | 1 | 93.81 | 3.66 | -6.39 | 0 | 0 | 5.98 |
| AI-160 | 384.51 | 28 | 2 | 4 | 0 | 60.44 | 3.85 | -5.76 | 0 | 0 | 5.41 |
| AI-193 | 452.67 | 33 | 3 | 3 | 0 | 43.37 | 6.16 | -4.17 | 1 | 0 | 5.86 |
| AI-198 | 482.61 | 35 | 4 | 6 | 0 | 78.9 | 4.37 | -5.73 | 0 | 0 | 6.41 |
| AI-59 | 408.53 | 30 | 1 | 4 | 1 | 67.51 | 3.98 | -5.77 | 0 | 0 | 5.71 |
| AI-103 | 424.70 | 31 | 4 | 1 | 0 | 17.07 | 7.53 | -2.63 | 1 | 0 | 6.11 |
| AI-128 | 454.60 | 33 | 3 | 5 | 1 | 76.74 | 4.38 | -5.84 | 0 | 0 | 6.02 |
| AI-138 | 484.67 | 35 | 5 | 5 | 0 | 61.83 | 5.10 | -5.15 | 1 | 0 | 6.55 |
| AI-161 | 456.61 | 33 | 3 | 5 | 1 | 72.83 | 4.20 | -5.62 | 0 | 0 | 6.21 |
| AI-24 | 470.64 | 34 | 4 | 5 | 0 | 61.83 | 4.81 | -5.32 | 1 | 0 | 6.42 |
| AI-116 | 466.57 | 34 | 3 | 6 | 0 | 86.74 | 3.80 | -6.26 | 0 | 0 | 6.11 |
| AI-117 | 466.57 | 34 | 3 | 6 | 1 | 93.81 | 3.67 | -6.39 | 0 | 0 | 5.98 |
| AI-142 | 426.72 | 31 | 4 | 1 | 1 | 20.23 | 7.42 | -2.24 | 1 | 0 | 6.21 |
| AI-157 | 384.51 | 28 | 2 | 4 | 0 | 60.44 | 3.89 | -5.76 | 0 | 0 | 5.41 |
| AI-169 | 470.68 | 34 | 2 | 4 | 1 | 59.06 | 5.29 | -5.13 | 1 | 0 | 6.58 |
| AI-185 | 424.53 | 31 | 1 | 5 | 2 | 87.74 | 3.25 | -6.55 | 0 | 0 | 5.8 |
| AI-180 | 468.58 | 34 | 3 | 6 | 1 | 89.9 | 3.87 | -6.03 | 0 | 0 | 6.21 |
| AI-203 | 386.48 | 28 | 2 | 5 | 0 | 69.67 | 3.35 | -6.34 | 0 | 0 | 5.43 |
| AI-61 | 412.69 | 30 | 5 | 1 | 1 | 20.23 | 6.98 | -2.74 | 1 | 0 | 6.21 |
| AI-65 | 538.67 | 39 | 5 | 7 | 1 | 99.13 | 4.39 | -5.74 | 1 | 0 | 6.85 |
| AI-75 | 454.60 | 33 | 3 | 5 | 1 | 72.83 | 4.03 | -6.15 | 0 | 0 | 6.2 |
| AI-91 | 452.58 | 33 | 3 | 5 | 1 | 76.74 | 4.17 | -5.70 | 0 | 0 | 6.04 |
| AI-94 | 452.58 | 33 | 3 | 5 | 1 | 76.74 | 4.27 | -5.70 | 0 | 0 | 6.04 |
| AI-115 | 484.62 | 35 | 6 | 6 | 0 | 86.74 | 3.90 | -6.40 | 0 | 0 | 6.34 |
| AI-131 | 498.61 | 36 | 4 | 7 | 1 | 99.13 | 3.54 | -6.52 | 0 | 0 | 6.56 |
| AI-140 | 484.58 | 35 | 3 | 7 | 2 | 110.13 | 3.03 | -6.82 | 0 | 0 | 6.37 |
| AI-150 | 468.58 | 34 | 3 | 6 | 1 | 89.9 | 3.75 | -6.35 | 0 | 0 | 6.19 |
| AI-164 | 450.57 | 33 | 3 | 5 | 0 | 73.58 | 4.34 | -5.63 | 0 | 0 | 5.89 |
| AI-188 | 454.60 | 33 | 3 | 5 | 0 | 69.67 | 4.54 | -5.51 | 1 | 0 | 5.96 |
| AI-27 | 452.58 | 33 | 3 | 5 | 0 | 73.58 | 4.42 | -5.60 | 0 | 0 | 5.82 |
| AI-82 | 436.58 | 32 | 3 | 4 | 0 | 56.51 | 5.00 | -4.90 | 1 | 0 | 5.87 |
| AI-110 | 400.51 | 29 | 2 | 5 | 1 | 80.67 | 3.04 | -6.55 | 0 | 0 | 5.62 |
| AI-175 | 484.58 | 35 | 3 | 7 | 2 | 110.13 | 3.00 | -7.14 | 0 | 0 | 6.35 |
| AI-189 | 440.70 | 32 | 4 | 2 | 1 | 37.3 | 6.53 | -3.63 | 1 | 0 | 6.05 |
| AI-190 | 512.64 | 38 | 4 | 5 | 0 | 73.58 | 5.38 | -4.83 | 2 | 0 | 6.01 |
| AI-36 | 526.70 | 38 | 4 | 6 | 1 | 85.36 | 4.73 | -5.96 | 1 | 0 | 6.91 |
| AI-47 | 436.58 | 32 | 3 | 4 | 0 | 56.51 | 5.06 | -4.90 | 1 | 0 | 5.87 |
| AI-71 | 512.63 | 37 | 5 | 7 | 1 | 99.13 | 4.10 | -6.54 | 1 | 0 | 6.54 |
| AI-133 | 414.71 | 30 | 6 | 1 | 1 | 20.23 | 7.19 | -2.2 | 1 | 0 | 6.3 |
| AI-166 | 400.51 | 29 | 2 | 5 | 1 | 80.67 | 3.10 | -6.55 | 0 | 0 | 5.62 |
| AI-178 | 454.60 | 33 | 3 | 5 | 1 | 72.83 | 4.09 | -6.15 | 0 | 0 | 6.2 |
| AI-30 | 386.65 | 28 | 5 | 1 | 1 | 20.23 | 6.75 | -2.47 | 1 | 0 | 5.98 |
| AI-37 | 472.70 | 34 | 2 | 4 | 2 | 66.76 | 5.07 | -5.31 | 1 | 0 | 6.35 |
| AI-78 | 482.57 | 35 | 3 | 7 | 1 | 103.04 | 3.77 | -6.51 | 0 | 0 | 6.09 |
| AI-96 | 466.57 | 34 | 3 | 6 | 1 | 93.81 | 4.05 | -5.81 | 0 | 0 | 6.14 |
| AI-121 | 440.57 | 32 | 3 | 5 | 1 | 76.74 | 4.08 | -6.14 | 0 | 0 | 5.85 |
| AI-165 | 466.57 | 34 | 3 | 6 | 1 | 93.81 | 3.66 | -6.39 | 0 | 0 | 5.98 |
| AI-40 | 454.60 | 33 | 3 | 5 | 1 | 76.74 | 4.38 | -5.84 | 0 | 0 | 6.02 |
| AI-35 | 400.51 | 29 | 2 | 5 | 1 | 80.67 | 3.04 | -6.55 | 0 | 0 | 5.62 |
| AI-45 | 484.58 | 35 | 3 | 7 | 2 | 110.13 | 2.75 | -7.14 | 0 | 0 | 6.35 |
| AI-62 | 500.58 | 36 | 3 | 8 | 3 | 130.36 | 2.44 | -7.75 | 1 | 0 | 6.67 |
| AI-64 | 496.59 | 36 | 3 | 7 | 2 | 114.04 | 3.01 | -7.04 | 0 | 0 | 6.34 |
| AI-72 | 440.70 | 32 | 4 | 2 | 1 | 37.3 | 6.50 | -3.63 | 1 | 0 | 6.05 |
| AI-88 | 312.40 | 23 | 2 | 3 | 0 | 43.37 | 4.07 | -5.32 | 0 | 0 | 3.77 |
| AI-109 | 454.60 | 33 | 3 | 5 | 1 | 72.83 | 4.10 | -6.15 | 0 | 0 | 6.2 |
| AI-136 | 452.58 | 33 | 3 | 5 | 1 | 76.74 | 4.25 | -5.7 | 0 | 0 | 6.04 |
| AI-154 | 300.39 | 22 | 1 | 3 | 1 | 54.37 | 3.83 | -4.77 | 0 | 0 | 3.38 |
| AI-176 | 456.61 | 33 | 3 | 5 | 1 | 72.83 | 4.21 | -5.62 | 0 | 0 | 6.21 |
| AI-76 | 544.72 | 39 | 4 | 7 | 3 | 113.29 | 3.89 | -6.38 | 1 | 0 | 6.97 |
| AI-93 | 528.72 | 38 | 5 | 6 | 2 | 93.06 | 4.65 | -5.77 | 1 | 0 | 6.97 |

CN = Compound number, MW = Molecular weight, HA = Heavy atoms, RB = Rotatable bonds, HBA = Hydrogen bond acceptors, HBD = Hydrogen bond donors, TPSA = Topical polar surface area (Å²), Log P = Lipophilicity, SP = Skin permeation log Kp (cm/s), LV = Lipinski violations, PAINS = PAINS alerts, SA = Synthetic accessibility

**Table S4. Potential human protein targets for inhibitors of the DENV-2 capsid protein, identified through computational analysis using SwissTargetPrediction.**

| **Compound** | **Off-target** | **Target class** | **Probability** |
| --- | --- | --- | --- |
| AI-149 | Glycogen synthase kinase-3 beta | Kinase | 1.00 |
| AI-61 | Niemann-Pick C1-like protein 1 | Other membrane protein | 0.89 |
|  | LXR-alpha | Nuclear receptor | 0.59 |
|  | Nuclear receptor ROR-gamma | Nuclear receptor | 0.56 |
| AI-65 | Niemann-Pick C1-like protein 1 | Other membrane protein | 0.89 |
|  | LXR-alpha | Nuclear receptor | 0.59 |
|  | Nuclear receptor ROR-gamma | Nuclear receptor | 0.56 |
| AI-140 | Glycogen synthase kinase-3 beta | Kinase | 0.97 |
| AI-30 | HMG-CoA reductase | Oxidoreductase | 0.68 |
|  | Cytochrome P450 51 | Cytochrome P450 | 0.68 |
|  | Androgen receptor | Nuclear receptor | 0.65 |
|  | Niemann-Pick C1-like protein 1 | Membrane protein | 0.65 |
|  | LXR-alpha | Nuclear receptor | 0.62 |
|  | Cytochrome P450 17A1 | Cytochrome P450 | 0.51 |
| AI-96 | Niemann-Pick C1-like protein 1 | Membrane protein | 0.89 |
|  | Nuclear receptor ROR-gamma | Nuclear receptor | 0.79 |
|  | Nuclear receptor subfamily 1 group H member 3 | Nuclear receptor | 0.79 |
